# Admixture Mapping of Sepsis in European Individuals With African Ancestries

**DOI:** 10.3389/fmed.2022.754440

**Published:** 2022-03-08

**Authors:** Tamara Hernandez-Beeftink, Itahisa Marcelino-Rodríguez, Beatriz Guillen-Guio, Héctor Rodríguez-Pérez, Jose M. Lorenzo-Salazar, Almudena Corrales, Ana Díaz-de Usera, Rafaela González-Montelongo, David Domínguez, Elena Espinosa, Jesús Villar, Carlos Flores

**Affiliations:** ^1^Research Unit, Hospital Universitario Nuestra Señora de Candelaria, Universidad de La Laguna, Santa Cruz de Tenerife, Spain; ^2^Research Unit, Hospital Universitario de Gran Canaria Dr. Negrín, Las Palmas de Gran Canaria, Spain; ^3^Genomics Division, Instituto Tecnológico y de Energías Renovables (ITER), Santa Cruz de Tenerife, Spain; ^4^CIBER de Enfermedades Respiratorias, Instituto de Salud Carlos III, Madrid, Spain; ^5^Department of Anesthesiology, Hospital Universitario Nuestra Señora de Candelaria, Santa Cruz de Tenerife, Spain

**Keywords:** sepsis, susceptibility, local ancestry, European, polymorphism

## Abstract

Sepsis is a severe systemic inflammatory response to infections that is accompanied by organ dysfunction. Although the ancestral genetic background is a relevant factor for sepsis susceptibility, there is a lack of studies using the genetic singularities of a recently admixed population to identify loci involved in sepsis susceptibility. Here we aimed to discover new sepsis loci by completing the first admixture mapping study of sepsis in Canary Islanders, leveraging their distinctive genetic makeup as a mixture of Europeans and African ancestries. We used a case-control approach and inferred local ancestry blocks from genome-wide data from 113,414 polymorphisms genotyped in 343 patients with sepsis and 410 unrelated controls, all ascertained for grandparental origin in the Canary Islands (Spain). Deviations in local ancestries between cases and controls were tested using logistic regressions, followed by fine-mapping analyses based on imputed genotypes, *in silico* functional assessments, and gene expression analysis centered on the region of interest. The admixture mapping analysis detected that local European ancestry in a locus spanning 1.2 megabases of chromosome 8p23.1 was associated with sepsis (lowest *p* = 1.37 × 10^−4^; Odds Ratio [OR] = 0.51; 95%CI = 0.40–0.66). Fine-mapping studies prioritized the variant rs13249564 within intron 1 of *MFHAS1* gene associated with sepsis (*p* = 9.94 × 10^−4^; OR = 0.65; 95%CI = 0.50–0.84). Functional and gene expression analyses focused on 8p23.1 allowed us to identify alternative genes with possible biological plausibility such as defensins, which are well-known effector molecules of innate immunity. By completing the first admixture mapping study of sepsis, our results revealed a new genetic locus (8p23.1) harboring a number of genes with plausible implications in sepsis susceptibility.

## Introduction

Sepsis is a severe systemic inflammatory response to an infection that is accompanied by organ dysfunction (1). It is the leading cause of death in adult intensive care units (ICUs) and is associated with a mortality rate of about 30% (2). Patients with sepsis may also develop severe respiratory complications, such as the acute respiratory distress syndrome (ARDS) (3), which further increases the risk of death and leads to disabling consequences for years in surviving patients. Multiple studies in animal models and in human cohorts support that the susceptibility to infections and the host immune response to infectious agents are strongly influenced by genetic variation (4–7). Genome-wide associations studies (GWAS) and small sized whole-exome sequencing studies have identified a few genetic variants associated with sepsis progression and outcome (8–10). Nevertheless, reproducible associations of genetic variants with sepsis susceptibility and outcomes are scarce (11), and further genome-wide assessments are needed.

Many studies have associated the ancestry of patients with the risk for sepsis and with poor sepsis outcomes (12–15). Furthermore, genetic ancestry has been associated with susceptibility to critical illnesses (16). In fact, some genetic loci show evident connections between genetic ancestry and susceptibility to infections (17, 18). In such a scenario, admixture mapping studies represent a more powered alternative than the traditional GWAS for the identification of disease genes in recently admixed populations. These analyses leverage the regional differences in the genetic ancestry blocks of megabase (Mb) size across the genome to detect disease loci that tend to be coinherited and their impact on the disease under study, requiring comparable smaller sample sizes to reach sufficient statistical power (19, 20). To date, no genetic ancestry studies have been conducted to identify sepsis risk genes.

The population of The Canary Islands, a Spanish archipelago in southwestern Europe, preserves a well characterized genetic admixture resulting from Europeans (EUR), North Africans (NAF), and sub-Saharan Africans (SSA). We have recently estimated their genetic ancestry proportions as 75% EUR, 22% NAF, and 3% SSA based on average genome-wide polymorphism data (21). Of note, the Canary Islanders exhibit the largest proportion of NAF ancestry among southwestern EUR populations described so far (21). Additionally, some genetic loci of this population present a large deviation in African and EUR ancestries, and those loci were found to be enriched in genes linked to infectious diseases, including infections causing ARDS (21). Some of these loci also harbored signatures of strong natural selection, including the human leukocyte antigen (HLA) system genes that are critically involved in the susceptibility to infectious diseases (17, 22).

Based on the described evidences and the key biomedical implications of NAF ancestry in EUR populations (23), we hypothesize that the distinctive genetic makeup of The Canary Islands population offers a powerful opportunity to leverage genetic ancestry for identifying sepsis susceptibility genes. To assess this possibility, here we describe the results of the first admixture mapping study of sepsis.

## Materials and Methods

### Samples, Genotyping, and Reference Population Datasets

This study was approved by the Research Ethics Committee of the participating center (Hospital Universitario Nuestra Señora de Candelaria: CHUNSC_2018-16). We obtained written informed consent from all participants or an appropriate proxy. We used a case-control approach with DNA samples from 763 subjects. Controls included 416 individuals from the Cardiovascular, Diabetes and Cancer cohort study (24). All individuals declared at least two generations of ancestors born in the Canary Islands, as has been described elsewhere (21). A total of 347 patients with sepsis from the GEN-SEP study admitted into a network of Spanish ICUs, with at least two generations of ancestors born in the Canary Islands, were used as cases. Sepsis was defined according to the Third International Consensus Definitions for Sepsis (1) (see Supplementary Methods in Supplementary Material for details).

The Axiom® Genome-Wide Human CEU 1 Array (Thermo Fisher Scientific, Waltham, MA) was used for genotyping 587,352 variants in DNA from donors with the support of the National Genotyping Center (CeGen), Universidad de Santiago de Compostela Node (Spain). Quality control procedures of data were performed with R 3.2.2 and PLINK v1.07 (25). Samples with a genotype call rate <95% or family relationships (PIHAT>0.2) with others were removed, resulting in a total of 753 individuals for further analyses, (343 were patients with sepsis and 410 population controls). Moreover, SNP filtering based on genotyping rate <95%, minor allele frequency (MAF) <0.01, and large deviations from Hardy-Weinberg equilibrium expectations (*p* < 1 × 10^−6^) left a total of 494,390 variants.

To obtain the ancestry estimates, and to maximize the intersection of autosomal SNPs in subsequent analyses from the datasets of cases, controls, and reference populations, we followed the methods described elsewhere (21). We extracted EUR and SSA datasets from the 1000 Genomes Project (1KGP) Phase 3 data (26). The NAF representation was gathered from 125 samples with origins in North and South Morocco, Western Sahara, Algeria, Tunisia, Egypt, and Libya that were previously genotyped with the Genome-Wide Human SNP Array 6.0 (Affymetrix, Santa Clara, CA) (27). Genotyping quality controls were performed using PLINK v1.07. EUR, NAF, and SSA individuals with genotype call rates <95% were removed from the analysis, as well as SNPs with >5% missing rate or deviating from the Hardy-Weinberg equilibrium expectations (*p* < 1 × 10^−6^) in each population. The intersection and post-filtering resulted in 113,414 SNPs genotyped for downstream analyses in cases, controls, and reference populations (see Supplementary Methods in Supplementary Material for details).

### Population Analyses

ADMIXTURE v1.3 (28) was used to estimate proxies for global ancestry proportions and verify that cases and controls were similar in these terms. Briefly, cross-validation error was lowest for k = 4, allowing to differentiate the SSA, NAF and EUR components clearly. The latter was detected as two separate ancestries (29) that were considered in aggregate in the analyses for simplicity. ADMIXTURE results were represented with CLUMPAK (30) (see Supplementary Methods in Supplementary Material for details).

### Local Ancestry and Association Analysis

Admixture mapping analyses were based on local ancestry block estimates across autosomes, which were inferred using Efficient Local Ancestry Inference (ELAI v1.0) (31), and assuming three admixing populations (EUR, NAF, and SSA) as has been described elsewhere (32). Association testing was performed with EPACTS v3.2.6 software (33) between local ancestry scores at each genomic position and sepsis susceptibility for each ancestry separately. Resulting p-values were corrected using a genomic control strategy based on λ calculation (34). The significance threshold was declared as *p* = 1.82 × 10^−4^ after Bonferroni correction for an average of 276 ancestry blocks, as has been inferred for this population (21). Regional plots of association results were represented with LocusZoom (35). The Power Analysis in Multiancestry Admixture Mapping (PAMAM) (36) was used to calculate the statistical power of the study. Based on PAMAM, assuming a design of 300 cases and 400 controls, a significance threshold of 1.82 × 10^−4^, and the known EUR admixture, this study achieved >80% power to detect an Odds Ratio (OR) <0.55 (Supplementary Figure 1).

We performed a fine-mapping study of the significant admixture mapping region. For this, SNP imputation of the whole chromosome containing the region was conducted with the Michigan Imputation Server tool (37) using the Haplotype Reference Consortium (HRC) version r1.1 2016 as the reference panel (38) and estimating haplotypes with Shape-IT v2.r790 (39). EPACTS v3.2.6 (33) was used to perform SNP association testing with allele dosage data of those variants with MAF >0.01 and imputation quality (Rsq) >0.3 lying in the region. A Bonferroni correction was performed to prioritize the most significant variant within the 1.2 Mb significant admixture mapping region. Based on a total of 449 independent tests examined in the region, significance was established at *p* < 1.57 × 10^−4^ (suggestive significance at *p* < 3.13 × 10^−3^). Conditional logistic regressions were assessed using R 3.6.0 to reveal the independent SNP associations near the significant admixture mapping region.

### Selective Sweep Analysis

We used iSAFE v1.0.4 (40) to provide evidence of a selective sweep embedded in the significant admixture mapping region and to pinpoint the most likely favored variant. iSAFE exploits the evolutionary contributions hidden in the flanking regions surrounding the region under selection to provide a ranking of variants (iSAFE-score) based on their contribution to the overall signal of selection.

For the analysis, we used phased data from 59 unrelated Iberians (IBS) from 1KGP and a random selection of 10 YRI subjects (out of the 108 available) to represent a non-target or outgroup population in GRCh37/hg19 coordinates. Data from chromosome 8p23.1 from 59 unrelated NAF samples were also used for comparisons. Since 1KGP does not include NAF datasets, we accessed data from whole-genome sequence from donors originating across NAF populations (Algeria [n = 3], Berber [n = 6], Egypt [n = 2], Libya [n = 2], Morocco [n = 8], Western Sahara [n = 4], Tunisia [n = 32], North Africa [undisclosed country, *n* = 2]). Seventeen of these samples were sequenced to a mean coverage >25X with Illumina HiSeq 2000 using 101 bases with paired end reads (41). Another 42 samples were sequenced using 150 paired-end reads either with Illumina HiSeq 4000 (*n* = 24, mean coverage >36X) or Illumina NovaSeq 6000 (*n* = 18, mean coverage >20X). Variant calling of this data was performed following best practices with an in-house pipeline based on the Burrows-Wheeler Alignment Tool (BWA-MEM v.0.7.12-r1039) and GATK v4 using the GRCh37/hg19 as a reference. Resulting calls were filtered to keep biallelic SNPs flagged as PASS. This callset was phased into haplotypes without providing any reference data using Eagle v2.4.1 (42).

iSAFE was executed in the region of interest enabling the IgnoreGaps flag and the default MaxFreq value (0.95). Ancestral fasta sequences for Homo sapiens (GRCh37) were downloaded from ENSEMBL release 75 (http://ftp.ensembl.org/pub/release-75/fasta/ancestral_alleles/). Given the exceptional performance of iSAFE in prioritizing the most likely favored variant in 94% of the times among the SNPs with the highest iSAFE scores (40), we performed the functional annotation of the 20 top ranked SNPs with the best scores using the Variant-to-Gene (V2G) pipeline aggregation from Open Target Genetics (43).

### *In silico* Functional and Gene Expression Analyses

To assess the functional role of the most significant variant and its best proxies (r^2^ >0.8), we accessed empirical data from different integrated online software tools, including LDLink v.4.1.0 webtool (44), The Open Targets Post-GWAS webtool (43), GTEx v.7 (45), HaploReg v4.1 (46), RegulomeDB v.2.0 (47), SNPDelScore (48), CHiCP (49), and the 3D Genome Browser (50). We also accessed three publicly available gene expression data, GSE32707 (51), GSE57065 (52, 53), and GSE28750 (54), from the Gene Expression Omnibus (GEO) data repository to assess differential expression of the genes residing in the significant admixture mapping region (plus 1 Mb on each side). The gene expression of all genes located within the region were compared using a Student's *t*-test. Finally, we performed a meta-analysis through a Fisher test of these GEO data sets using ImaGEO (55) (see Supplementary Methods in Supplementary Material for details).

## Results

### Admixture and Fine-Mapping

Basic clinical and demographical data of cases and controls are provided in Table 1. Further details are included in the supplementary material (Supplementary Results; Supplementary Table 1; Supplementary Figure 2).

**Table 1:** Relevant demographic and clinical features of study samples analyzed after quality control procedures.

	**Sepsis cases** **(*n* = 343)**	**Controls** **(*n* = 410)**	*p*-value^*^
Gender (% male)^*^	67	49	<0.0001
Age (years, mean ± SD)^#^	61 ± 15	42 ± 13	<0.0001
BMI (kg/m^2^, mean ± SD)^#^	28 ± 8	27 ± 5	0.835

* *Gender comparison was conducted by chi-square test.*

# *Age and body mass index (BMI) were compared using the Mann–Whitney U-test*.

**Figure 1:**
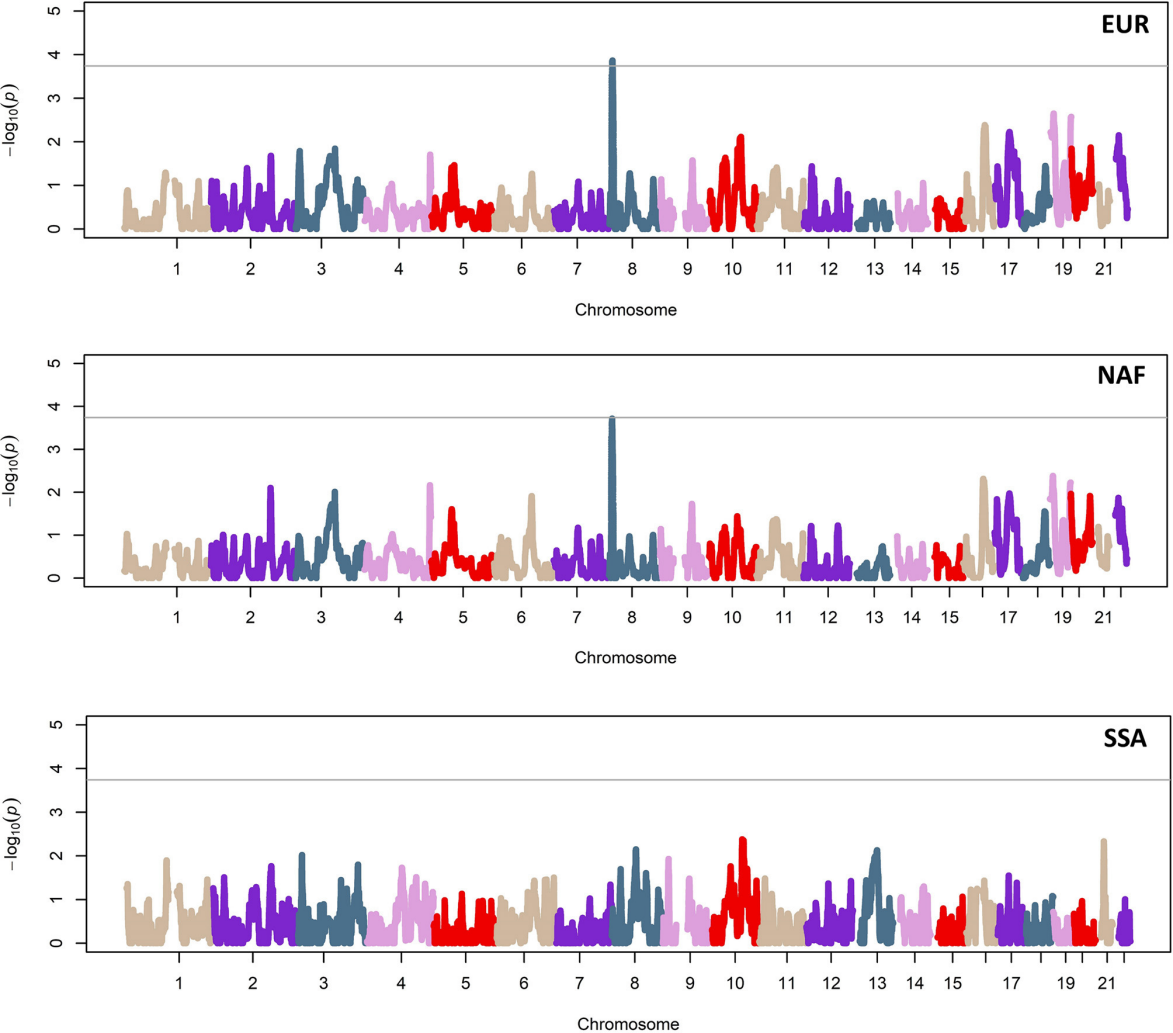
Manhattan plots of the admixture mapping study based on local ancestry estimates of European (EUR; top), North African (NAF; middle), and sub-Saharan African (SSA; bottom) populations. Horizontal lines indicate the genome-wide significance threshold (*p* = 1.82 × 10^−4^).

The admixture mapping study was performed using EUR, NAF, and SSA local ancestry estimates for 113,414 variant positions across the genomes of all cases and controls. No inflation effects were identified in the association results for any ancestry (λEUR = 0.96, λNAF = 0.96, and λSSA = 0.99). Association testing of the three ancestry scores with sepsis susceptibility (Figure 1) revealed 114 consecutive positions at 8p23.1 (chr8: 8,155,475–9,318,404; GRCh37/hg19 coordinates) associated with sepsis protection. The strongest significance was obtained for rs17149618 (*p* = 1.37 × 10^−4^; OR [95%CI] = 0.51 [0.40–0.66]) and it was related to a single local EUR ancestry peak of significance (Figure 1; Supplementary Figure 3; Supplementary Table 2). For rs17149618, the NAF ancestry associated with sepsis risk (*p* = 1.93 × 10^−4^; OR [95%CI] = 2.01 [1.57–2.58]). The SSA ancestry blocks were unrelated with this result and lacked significant associations with sepsis in the study population (Figure 1).

**Figure 2:**
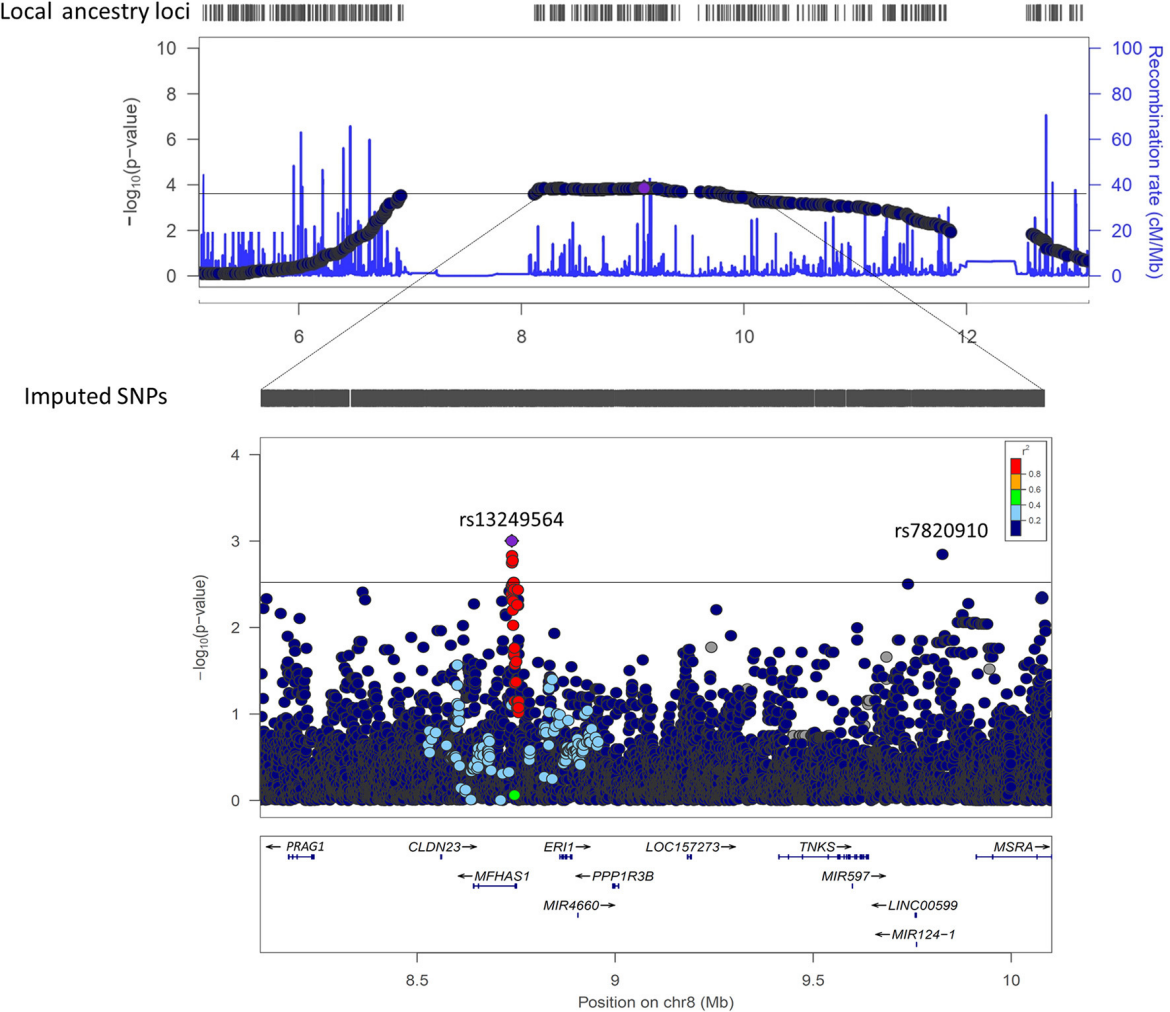
Regional plots from admixture (top) and fine-mapping (bottom) studies at 8p23.1, showing the -log10(p-value) transformed significance (y-axis) vs. genomic position (x-axis). In the admixture mapping plot, the estimated recombination rates (light blue curve) are plotted on the right y-axis. The horizontal line indicates the significance threshold (*p* = 1.82 × 10^−4^). In the fine-mapping plot, the results indicate the leading SNP and the results for the remaining SNPs are color coded to reflect their degree of linkage disequilibrium with it based on pairwise r^2^ values in European populations. The horizontal line indicates the suggestive threshold (*p* = 3.13 × 10^−3^).

The region of 8p23.1 associated with sepsis in this study is broad (~1.2 Mb) and harbors several genes. In particular, most of the 114 significant positions were intergenic, although 27 of them corresponded to positions of the genes encoding the Malignant Fibrous Histiocytoma Amplified Sequence 1 (*MFHAS1*), Exoribonuclease 1 (ERI1), and PEAK1 Related, Kinase-Activating Pseudokinase 1 (*PRAG1*) (Supplementary Table 2). Strikingly, we noted a region telomeric to the significant admixture mapping region of 8p23.1 showing a gap of mapped genetic variation (Figure 2) that we interpreted as a consequence of repetitive elements associated with the cluster of defensin genes mapping in the region (*DEFB103B, DEFB103A, DEFB109P1B, DEFB4A, DEFB4B*, and *DEFB130*). Therefore, this admixture mapping study was unable to assess the genetic variation of these key effector molecules of innate immunity (55).

A joint analysis model of the local ancestry estimates and allele dosages at the most significant locus (rs17149618) had a very subtle impact on the EUR ancestry association (*p* = 4.11 × 10^−5^; OR [95%CI] = 0.46 [0.27–0.78]) (Table 2), suggesting that genetic variation at rs17149618 does not explain this admixture mapping peak. A fine-mapping assessing the genetic association of SNP variants of the 8p23.1 region was then performed to test other nearby variants as alternative explanations of the admixture mapping result (Figure 2). This analysis revealed two independent genetic variants with a suggestive association with sepsis protection, the most significant variant located within intron 1 of *MFHAS1* (rs13249564; *p* = 9.94 × 10^−4^; OR = 0.65; 95%CI = 0.50–0.84) and an intergenic variant located between LINC00599 and *MSRA* (rs7820910; *p* = 1.42 × 10^−3^; OR = 0.49; 95%CI = 0.31–0.76) (Figure 2). A joint analysis of rs13249564 and local EUR ancestry did not alter the significance of the association of rs13249564 with sepsis (*p* = 2.20 × 10^−4^, OR [95% CI] = 0.59[0.45–0.78]), suggesting an independence between rs13249564 and EUR ancestry (Table 2). As a nested analysis, we then compared the two independent genetic variants between the population controls and the patients with sepsis caused by Gram-positive (*N* = 86, including those that were polymicrobial with mixed Gram-positive) or separately with those with Gram-negative bacteria (*N* = 115, including those that were polymicrobial with mixed Gram-negative). While both were significantly associated with Gram-negative bacterial sepsis (rs13249564, *p* = 1.39 × 10^−3^, OR [95% CI] = 0.50[0.33–0.77]; and rs7820910, *p* = 0.028, OR [95%CI] = 0.46[0.23–0.92]), none of the two was associated with Gram-positive bacterial sepsis (rs13249564, *p* = 0.113, OR [95% CI] = 0.70[0.45–1.09]; and rs7820910, *p* = 0.364, OR [95% CI] = 0.74[0.39–1.42]).

**Table 2:** Joint SNP-ancestry analysis in the 8p23.1 region.

**Factor**	**OR (95% CI)**	***p*-value**
EUR ancestry (at rs17149618)	0.51 (0.40–0.66)	1.37 × 10^−4^
Allele dosage of rs17149618	0.94 (0.60–1.47)	0.792
EUR ancestry (conditioned on rs17149618 allele dosage)	0.46 (0.27–0.78)	4.11 × 10^−5^
Fine mapping top (rs13249564)	0.65 (0.50–0.84)	9.94 × 10^−4^
Allele dosage of rs13249564 (conditioned on EUR ancestry)	0.59 (0.45–0.78)	2.20 × 10^−4^
Allele dosage of rs17149618 (conditioned on rs13249564)	0.95 (0.61–1.50)	0.835

*CI, confidence interval; EUR, European; OR, odds ratio; SNP, single nucleotide polymorphism*.

**Figure 3:**
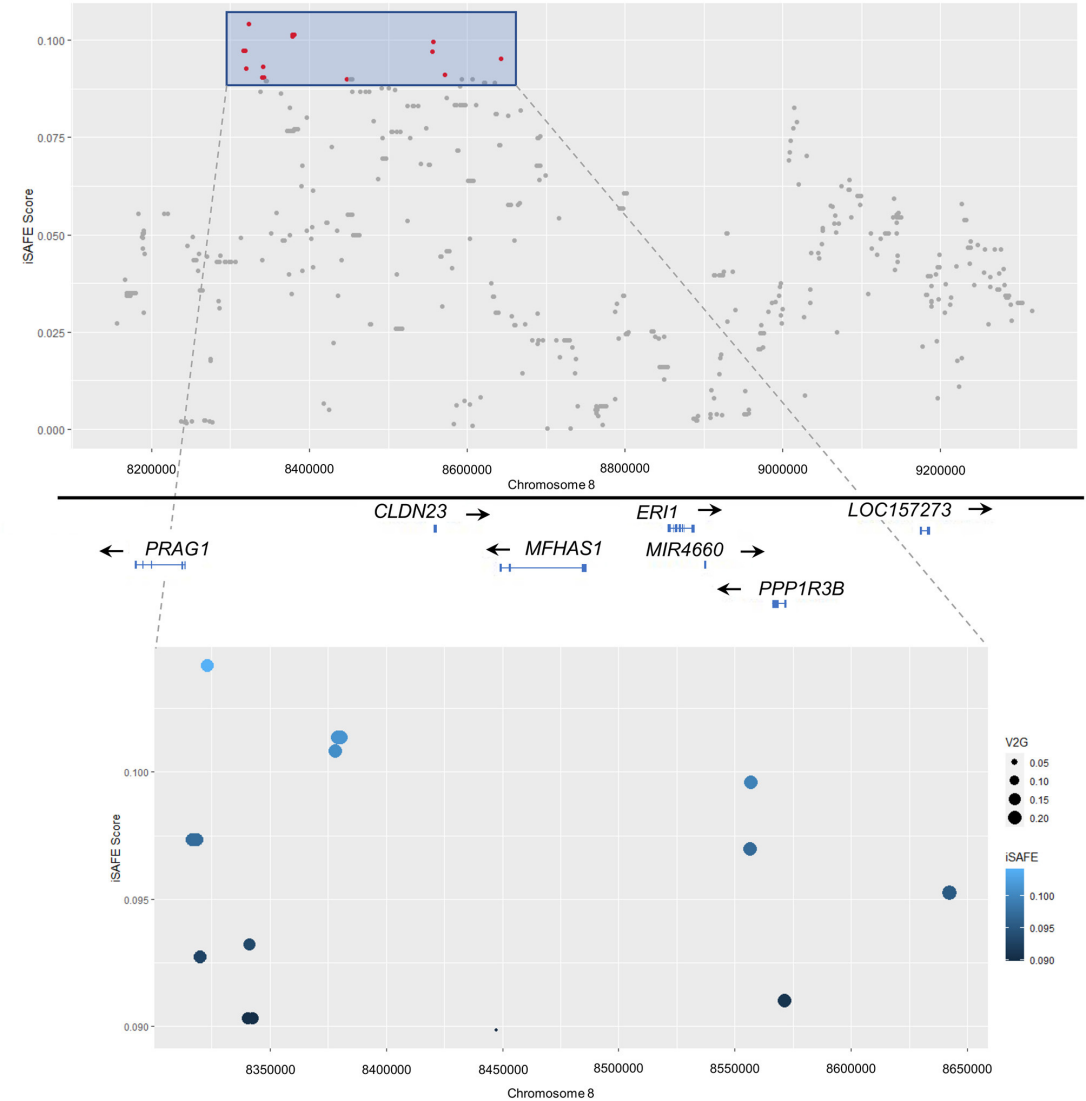
Plot representing the iSAFE score for the SNPs on the significant admixture mapping region at 8p23.1 (top) and the 20 SNPs with the higher iSAFE scores and their functional annotation using the Variant-to-Gene (V2G) pipeline aggregation and scoring (bottom). Both plots show iSAFE scores (y-axis) vs. genomic position (x-axis).

Furthermore, to evidence if a selective sweep was embedded in the admixture mapping region, an iSAFE scan was performed. The iSAFE scores in NAF ranged from 4.55 × 10^−4^ to 0.04, whereas in the IBS scan the results ranged from 2.05 × 10^−4^ to 0.104. Thus, the iSAFE scores in the region were highest for the IBS, with the top-ranking variants corresponding to positions 8.3–8.6 Mb (iSAFE score≥0.09), near *PRAG1* and Claudin 23 (*CLDN23*) coding genes and telomeric to *MFHAS1* (Figure 3; Table 3).

**Table 3:** Functional annotation of the 20 SNPs with top iSAFE scores on IBS from the significant admixture mapping region.

**Position**	**RSID**	**REF**	**ALT**	**Nearest gene**	**Nearest coding gene**	**Freq EUR**	**CADD Scaled**	**eQTLs**	**V2G—*MFHAS1***
*8316637*	rs2921064	C	T	AC103957.2	*PRAG1*	0.519	2.280	125	0.181
*8317615*	rs2921061	A	T	AC103957.2	*PRAG1*	0.491	0.688	122	0.181
*8317817*	rs2921060	A	C	AC103957.2	*PRAG1*	0.491	2.970	121	0.181
*8318095*	rs2980766	T	C	AC103957.2	*PRAG1*	0.491	0.482	122	0.181
*8318667*	rs2921057	C	T	AC103957.2	*PRAG1*	0.509	6.070	121	0.181
*8320104*	rs2921051	C	A	AC103957.2	*PRAG1*	0.491	0.925	124	0.181
*8323088*	rs2979181	A	T	AC103957.2	*PRAG1*	0.519	2.000	131	0.181
*8340477*	rs2921028	T	C	AC103957.2	*PRAG1*	0.462	0.156	119	0.163
*8341105*	rs2976902	T	C,G	AC103957.2	*PRAG1*	0.462	12.700	120	0.163
*8342415*	rs2976906	A	T	AC103957.2	*PRAG1*	0.462	0.103	119	0.163
*8378102*	rs13270194	T	C	AC114550.3	*PRAG1*	0.481	0.398	134	0.190
*8378992*	rs7837587	T	C	AC114550.3	*PRAG1*	0.481	0.430	135	0.190
*8379514*	rs7009054	T	C	AC114550.3	*PRAG1*	0.481	2.000	135	0.172
*8380471*	rs7827182	G	C	AC114550.3	*PRAG1*	0.472	1.680	135	0.190
*8447225*	rs190635314	G	C	AC114550.1	*CLDN23*	0.014	12.200	0	0.036
*8447226*	rs183370058	A	T	AC114550.1	*CLDN23*	0.014	13.300	0	0.036
*8556278*	rs28663303	T	C	*CLDN23*	*CLDN23*	0.425	0.609	88	0.209
*8556865*	rs68168815	G	C	*CLDN23*	*CLDN23*	0.425	0.967	92	0.209
*8571364*	rs1109618	A	T	*CLDN23*	*CLDN23*	0.406	0.157	91	0.209
*8642525*	rs4841039	G	A	AC087269.1	*CLDN23*	0.264	11.400	47	0.227

### Functional Assessments

When we explored the potential functional implications of the prioritized variant in the fine-mapping study, rs13249564, we observed high evidence that it is an important regulatory variant, featuring DNase QTL and expression QTL (eQTL) in several cell lines (Supplementary Table 3). A RegulomeDB score of 0.609 and a RegulomeDB category of 4 were determined for it, indicating a weak effect in a DNase I hypersensitive site and transcription factor binding site, respectively. We found that this variant has high CellulAr dePendent dEactivating (CAPE) scores for DNase QTL and eQTL in several cell lines (Supplementary Table 3). According to GTEx results, rs13249564 is an eQTL in 13 different tissues (Supplementary Table 3), in addition to 4,836 significant Single-Tissue eQTLs and 126 significant Single-Tissue splicing QTL for *MFHAS1* in all tissues. Likewise, there was also evidence of long-distance chromatin interactions among nearby candidate genes of the region, such as *ERI1, CLDN23* and Tankyrase (*TNKS*) genes in lymphoblastoid cells. On the other hand, some of its 44 proxies (r^2^ > 0.8) (Supplementary Figure 3) also have high CAPE scores for DNase QTL and eQTL in several cell lines, and many were significant eQTLs across different tissues in GTEx (Supplementary Table 4). Epigenome imputation, using peaks from H3K4me1 and H3K4me3 (as enhancers and promoters, respectively), and using peaks from H3K27ac and H3K9ac (as enhancers and promoters, respectively), revealed marks linked to most of the variants. Finally, we also annotated the functional implications of rs7820910, which also passed the suggestive threshold in the fine-mapping study (Figure 2). However, it lacked functional activity evidence (Supplementary Table 3).

### Gene Expression Analysis

The expression analysis of the genes mapping to the region 8p23.1 associated with sepsis revealed that besides *MFHAS1* and *CLDN23*, many of the top-most significant differential expression (q-value <0.001) between sepsis cases and controls were concentrated in defensin genes (*DEFA4* was the top ranked) (Supplementary Table 5). The results were similar in the three gene expression datasets comparing untreated and septic patients (GSE32707), healthy controls with those of septic shock patients (GSE57065), or with those from patients with sepsis (GSE28750).

## Discussion

To our knowledge, this is the first admixture mapping study of sepsis conducted to date. The Canary Islanders have a well-known distinctive genetic makeup reminiscent of the historical admixture between aboriginal populations from NAF, Southwestern EUR and SSA inhabitants (21). Many previous studies have provided evidence supporting a link between ancestry strata and sepsis susceptibility and outcomes (12–15). Besides, an admixture mapping study in African Americans identified that variation in the HLA class II region linked to EUR ancestry was associated with *Staphylococcus aureus* bacteremia (56). Our study revealed a link between local EUR ancestry in 8p23.1 with sepsis protection (NAF as a risk), supporting it as a novel sepsis locus. In agreement with this observation, our previous evolutionary genetic analysis in Canary Islanders supported that chromosome regions with outlier African or European ancestries were enriched in genes involved in infectious diseases (21). Since identifying the main drivers of associations in complex traits is challenging (57), we conducted a fine-mapping study, followed by a targeted gene expression analysis and SNP and gene-level functional assessments to prioritize the most likely gene driving the association in the 8p23.1 region with sepsis.

The fine mapping of the 8p23.1 region revealed a leading genetic variant significantly associated with sepsis located within intron 1 of the *MFHAS1* gene. Functional analysis showed that this variant and a few of its linkage disequilibrium (LD) proxies may have a potential regulatory implication, featuring DNase QTLs and eQTLs in several cell lines. Furthermore, we observed a significant overexpression of *MFHAS1* in peripheral blood of septic patients compared to ICU or healthy controls. Interestingly, MFHAS1 is a potential immune regulator dependent on Toll-like receptors (TLRs) (58), and has an important role in the inflammatory process (59). Zhong et al. (60) analyzed blood samples from septic patients after surgery and from patients undergoing selective surgery to determine changes in the MFHAS1 protein levels. They observed that the MFHAS1 protein increased during the immune response. Besides, *MFHAS1* gene expression was specially increased in peripheral blood mononuclear cells from patients with sepsis. In that study, MFHAS1 also showed dual effects on the TLR-2 signaling pathway and inflammation, with an inhibitory effect in the first few hours albeit with a stimulating effect after 24 h (60). Xu et al. (61) also explored whether MFHAS1 was involved in macrophage polarization, which is critical for balancing the innate immunity and the inflammatory injury by macrophages during sepsis (62), using *in vitro* pulldown assays and *in vivo* co-immunoprecipitation (63). They found that the ubiquitylation of MFHAS1 positively regulates TLR-2-mediated Jun NH2-terminal kinase (JNK)/p38 pathway and promotes M1 (pro-inflammatory) macrophage polarization, M2 to M1 macrophage transformation, and inflammation (63). MFHAS1 also acts as a suppressor of the TLR-4 signaling pathway and the inflammatory cytokine expression in cells exposed to lipopolysaccharide (64, 65). In agreement with this, the driver of variant associations was the sepsis originated by Gram-negative bacteria. Further studies, including *in vivo* and *in vitro* experiments, are needed to confirm this effect. Despite this, and irrespective of the fact that *MFHAS1* correlates with the lead variants in the admixture mapping stage, our results do not allow us to infer whether this gene is driving the association or its causally involved in sepsis. It could be speculated that the *MFHAS1* variants associated with the risk of sepsis (or variants in high LD) may affect gene activity by increasing *MFHAS1* expression, increasing inflammatory cytokines and cells of the immune system. Taken together, this evidence supports that *MFHAS1* deserves further exploration as a biological candidate gene for sepsis.

The functional and gene expression analyses on the 8p23.1 region also allowed us to alternatively hypothesize the role of defensins, with clear biological plausibility, as drivers of the admixture mapping results. Defensins have modulatory effects on both the innate and adaptive immune responses and represent a vital part of the human immune system due to their broad spectrum activity against pathogenic bacteria, fungi, protists, and enveloped viruses (66–68). For example, β-defensins (DEFBs) interact with TLR-4 receptors of immune cells and regulate the expression of inflammatory mediators through the Nuclear Factor Kappa B pathway (66, 69). DEFB-1 was previously studied in 211 patients with severe sepsis and 157 healthy controls (70). A candidate-gene case-control association study in a Chinese Han population showed that *DEFB1* genetic variants were associated with susceptibility to sepsis and poor outcomes, suggesting that *DEFB1* could be involved in the immune defense and inflammation response regulation during sepsis (70). In another study, the same group (71) also observed that *DEFA1/DEFA3* participate in host immune response to sepsis and its higher copy number variation was significantly associated with sepsis risk. In agreement with this, in a recent study, Chen et al. (72) showed in a mouse model of sepsis that animals with a higher copy number of *DEFA1*/*DEFA3* genes had more severe damage on vital organs and mortality than those with a lower copy number or the wild-type mice (72). Alpha defensins (DEFA4, DEFA5, and DEFA6) play an important role in regulation of bacterial colonization of the gut, as well as in the activation of pro- and anti-inflammatory response of the adaptive immune system cells (73). Taken together, these findings support the important roles of defensins in the development or outcomes of patients with sepsis. Therefore, we could not rule out the possibility that any of the defensin genes located in the admixture mapping region is driving the association with sepsis.

We acknowledge some limitations of our study. First, we have used controls that were not clinically characterized for sepsis or any respiratory diseases. We cannot rule out either that controls suffered sepsis at any time during their lives. We were unable to reduce the selection bias, neither controlling environmental risk factors in the selected controls. Despite that, such controls are widely used in many large-scale GWAS studies (74, 75). The inclusion of participants in this study was based on self-declaration of at least two generations of ancestors born in the Canary Islands. However, we do not think that there is any effect in our study results, because there is a high concordance with the genetic ancestry estimates for this population (21) and the self-declaration of ethnic categories is accepted by National Institutes of Health guidelines for medical and clinical research. Another limitation is the lack of a replication stage of our results in an independent study sample, motivated by the unavailability of genetic studies assessing populations with NAF ancestries as found the Canary Islanders (76). On the other hand, since clinical information in controls was not available, sensitivity analyses adjusting by relevant confounders could not be performed. Furthermore, due to the scarcity of reference genomic data from samples of NAF origin, the study relied on a limited number of genetic variants to estimate ancestry blocks. Finally, it is worth declaring the possibility that the content of the references for the imputation (clearly biased to represent diversity in European populations) may have decreased the power in the fine-mapping stage.

### Conclusions

In summary, we show the first admixture mapping study of sepsis. This allowed us to assess the association of local EUR ancestry with a protective effect against sepsis and suggested a sepsis risk association with local NAF ancestry at the 8p23.1 region, which harbors a novel promising genetic locus for sepsis susceptibility.

## Data Availability Statement

The datasets presented in this study can be found in online repositories. The names of the repository/repositories and accession number(s) can be found below: FigShare, 10.6084/m9.figshare.18393986.

## Ethics Statement

The studies involving human participants were reviewed and approved by Ethics Committee for Drug Research from the Hospital Universitario de Canarias (Code: CHUNSC_2018-16). The patients/participants provided their written informed consent to participate in this study.

## Author Contributions

CF conceived and designed research. TH-B, IM-R, BG-G, AC, RG-M, DD, EE, and CF performed experiments. TH-B, IM-R, BG-G, HR-P, JL-S, AD-dU, and CF analyzed data. TH-B, IM-R, BG-G, HR-P, JL-S, RG-M, JV, and CF interpreted results of experiments. TH-B and IM-R prepared figures. TH-B, IM-R, and CF drafted manuscript. TH-B, IM-R, BG-G, JL-S, AD-dU, RG-M, JV, and CF edited and revised manuscript. All authors contributed to the article and approved the submitted version.

## Funding

This study was funded by Instituto de Salud Carlos III (CB06/06/1088, PI14/00844, PI17/00610, FI17/00177, FI18/00230, CD19/00231, and PI20/00876) and co-financed by the European Regional Development Funds, a way of making Europe from the European Union; by Fundación CajaCanarias and Fundación Bancaria La Caixa (2018PATRI20); by Cabildo Insular de Tenerife (CGIEU0000219140); by the agreement OA17/008 with Instituto Tecnológico y de Energías Renovables (ITER) to strengthen scientific and technological education, training, research, development and innovation in Genomics, Personalized Medicine and Biotechnology; and by a fellowship from the Spanish Ministry of Education and Vocational Education (FPU16/01435).

## Conflict of Interest

The authors declare that the research was conducted in the absence of any commercial or financial relationships that could be construed as a potential conflict of interest.

## Publisher's Note

All claims expressed in this article are solely those of the authors and do not necessarily represent those of their affiliated organizations, or those of the publisher, the editors and the reviewers. Any product that may be evaluated in this article, or claim that may be made by its manufacturer, is not guaranteed or endorsed by the publisher.
