# Supplementary material for: Admixture Mapping of Sepsis in European Individuals With African Ancestries
Source: Front Med (Lausanne). 2022 Mar 8;9:754440. doi: 10.3389/fmed.2022.754440 (PMC8957104; doi:10.3389/fmed.2022.754440)
Supplement: Supplementary file 1 [file Data_Sheet_1.DOCX]

Supplementary Material

**Supplementary methods**

Samples

Controls included 416 individuals from the Cardiovascular, Diabetes and Cancer (CDC) cohort study (1). All these individuals were randomly recruited from 2000 to 2005, were aged between 18 and 75 years, and self-declared at least two generations of ancestors who were born in the Canary Islands, as has been described elsewhere (2). The GEN-SEP cohort is a national, multicenter, observational study conducted in a Spanish network of 11 intensive care units (ICUs) between 2002 and 2017. In this study we used a subset of 347 Canarian patients aged between 19 and 89 years. Peripheral blood was withdrawn at the time of inclusion into the study and stored at -20ºC until use. Illustra™ blood genomicPrep Mini Spin Kit (GE Healthcare) and Qubit 3.0 fluorometer (Thermo Fisher Scientific) were used for DNA extraction and quantification, respectively.

Reference population datasets and global admixtures analysis

We followed the methods described elsewhere (2) to maximize the intersection of autosomal SNPs from the data sets of cases, controls, and reference populations. We extracted EUR and SSA datasets from the 1000 Genomes Project (1KGP) Phase 3 data (3). Given that NAF ancestry is clearly distinguishable from that of Near Western influences in European populations (4), we used British, Finnish and Utah residents with Northern and Western European ancestry (overall n=289), as well as Yoruba Nigerians (n=108), as the optimal representatives for EUR and SSA sources, respectively. The NAF representation (n=125) was gathered from samples with origins in North and South Morocco, Western Sahara, Algeria, Tunisia, Egypt and Libya that were previously genotyped with the Genome-Wide Human SNP Array 6.0 (Affymetrix) (5). We also run a model-based analysis including data from reference populations from EUR, NAF, and SSA to verify that cases and controls were similar in terms of global genetic admixtures. In accordance with previous assessments and the best fitting number of ancestry partitions (k=4) (2), the identified clusters revealed NAF (22% in cases and controls), SSA (2% in cases, 3% in controls), and EUR (76% in cases, 75% in controls) ancestry components in the study sample (Supplement Figure S2). Global ancestry differences between cases and controls were subtle, strongly supporting an effective ancestry matching among samples.

*In silico* functional analyses

The Open Targets Post-GWAS webtool (6) was used to determine the regulatory effect of non-coding variants among the significant admixture mapping region. Likewise, different integrated online software tools were accessed to analyze the functional role of the most significant variant and its best proxies (r^2^ >0.8). We used the GTEx project dataset v.7 to assess local expression quantitative trait loci (eQTLs) (7), HaploReg v4.1 to predict potential regulatory genomic regions (8), and RegulomeDB v.2.0 to rank SNPs according to their potential functional roles (9). We also relied on the RegulomeDB probability score (ranging from 0 to 1, with 1 indicating high probability of a regulatory variant), which aggregates diverse functional genomics features along with continuous values such as ChIP-seq signals, DNase-seq signals, information content changes, and DeepSEA scores, among others. SNPDelScore was also used to quantify the deleterious effects of noncoding variants using a range of scores calculated for 44 independent cell lines (10).

Gene expression analyses

We accessed public gene expression data (GSE32707) (11) from the GEO data repository to assess differential blood gene expression between sepsis patients and ICU controls collected at inclusion and seven days later. A two-sample t-test was used to test significance, considering a False Discovery Rate (q-value) of 1% to limit false positives because of the multiple comparisons.

We also assessed two additional gene expression microarray data sets, GSE57065 (12) (13) and GSE28750 (14), for validation according to the procedures described above. We first assessed differential blood gene expression between a total of 25 healthy volunteers and 28 ICU patients at the onset of septic shock (GSE57065). Additionally, we evaluated blood gene expression data from hospital staff with no known concurrent illnesses against those from 27 critical patients with sepsis (GSE28750). Finally, we performed a meta-analysis through a Fisher test of these GEO data set results using ImaGEO (15). Results for all genes analyzed in the region are shown in Supplement Table S5.

Supplementary References

1. De León AC, Pérez MCR, González DA, Coello SD, Jaime AA, et al. Presentation of the "CDC de Canarias" cohort: objectives, design and preliminary results. Rev Esp Salud Publica (2008) 82(5):519-34. doi: 10.1590/s1135-57272008000500007.
2. Guillen-Guio B, Lorenzo-Salazar JM, González-Montelongo R, Díaz-de Usera A, Marcelino-Rodríguez I, et al. Genomic analyses of human European diversity at the southwestern edge: Isolation, African influence and disease associations in the Canary Islands. Mol. Biol. Evol. (2018) 35(12):3010–3026. doi: 10.1093/molbev/msy190.
3. Sudmant PH, Rausch T, Gardner EJ, Handsaker RE, Abyzov A, et al. An integrated map of structural variation in 2,504 human genomes. Nature (2015) 526(7571):75–81. doi: 10.1038/nature15394.
4. Botigue LR, Henn BM, Gravel S, Maples BK, Gignoux CR et al. Gene flow from North Africa contributes to differential human genetic diversity in southern Europe. Proc. Natl. Acad. Sci. (2013) 110(29):11791–11796. doi: 10.1073/pnas.1306223110.
5. Henn BM, Botigué LR, Gravel SR, Wang W, Brisbin A, et al. Genomic ancestry of North Africans supports back-to-Africa migrations. PLoS Genet. (2012) 8(1): e1002397. doi: 10.1371/journal.pgen.1002397.
6. Peat G, Jones W, Nuhn M, Marugán JC, Newell W, et al. The open targets post-GWAS analysis pipeline. Bioinformatics (2020) 36(9):2936–2937. doi: 10.1093/bioinformatics/btaa020.
7. Lonsdale J, Thomas J, Salvatore M, Phillips R, Lo E, et al. The Genotype-Tissue Expression (GTEx) project. Nat. Genet. (2013) 45(6):580–585. doi: 10.1038/ng.2653.
8. Ward LD and Kellis M. HaploReg: A resource for exploring chromatin states, conservation, and regulatory motif alterations within sets of genetically linked variants. Nucleic Acids Res. (2012) 40(D1):930–934. doi: 10.1093/nar/gkr917.
9. Boyle AP, Hong EL, Hariharan M, Cheng Y, Schaub MA, et al. Annotation of functional variation in personal genomes using RegulomeDB. Genome Res. (2012) 22(9):1790–1797. doi: 10.1101/gr.137323.112.
10. Alvarez RV, Li S, Landsman D, and Ovcharenko I. SNPDelScore: Combining multiple methods to score deleterious effects of noncoding mutations in the human genome. Bioinformatics (2018) 34(2):289–291. doi: 10.1093/bioinformatics/btx583.
11. Dolinay T, Kim YS, Howrylak J, Hunninghake GM, An CH et al. Inflammasome-regulated cytokines are critical mediators of acute lung injury. Am. J. Respir. Crit. Care Med. (2012) 185(11):1225–1234. doi: 10.1164/rccm.201201-0003OC.
12. Cazalis M-A, Lepape A, Venet F, Frager F, Mougin B, et al. Early and dynamic changes in gene expression in septic shock patients: a genome-wide approach. Intensive Care Med. Exp. (2014) 2(1):1–17. doi: 10.1186/s40635-014-0020-3.
13. Tabone O, Mommert M, Jourdan C, Cerrato E, Legrand M, et al. Endogenous retroviruses transcriptional modulation after severe infection, trauma and burn. Front. Immunol. (2019) 10:1–12. doi: 10.3389/fimmu.2018.03091.
14. Sutherland A, Thomas M, Brandon RA, Brandon RB, Lipman J, et al. Development and validation of a novel molecular biomarker diagnostic test for the early detection of sepsis. Crit. Care (2011) 15(3):R149. doi: 10.1186/cc10274.
15. Toro-Domínguez D, Martorell-Marugán J, López-Domínguez R, García-Moreno A, González-Rumayor V, et al. ImaGEO: Integrative gene expression meta-analysis from GEO database. Bioinformatics (2019) 35(5):880–882. doi: 10.1093/bioinformatics/bty721.

**Supplementary** **figures**


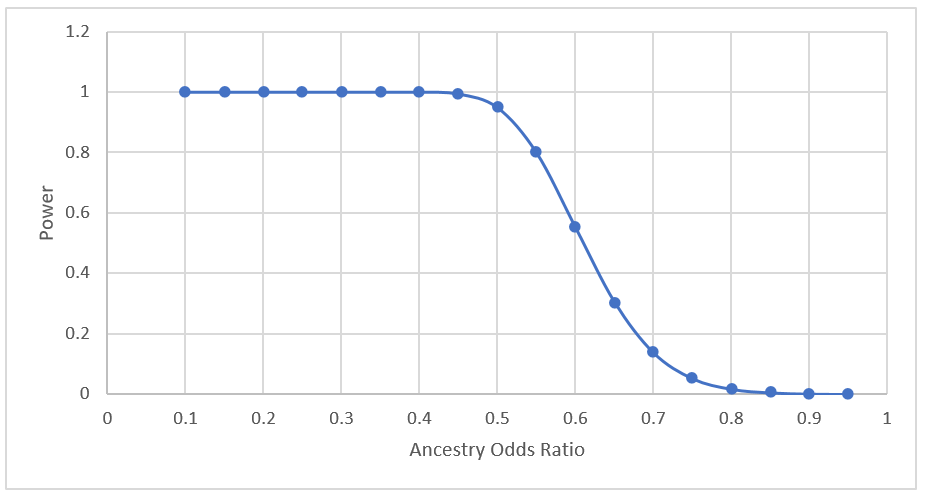


**Figure S1.** PAMAM power estimated for a range of ancestry effect sizes in a design of 300 cases and 400 controls. The assessment assumed a target significance threshold of p<1.82x10-4.

**Figure S2.** Admixture estimates (k=4) for sepsis cases, population controls, and Europeans (EUR), North Africans (NAF), and sub-Saharan Africans (SSA) for reference.


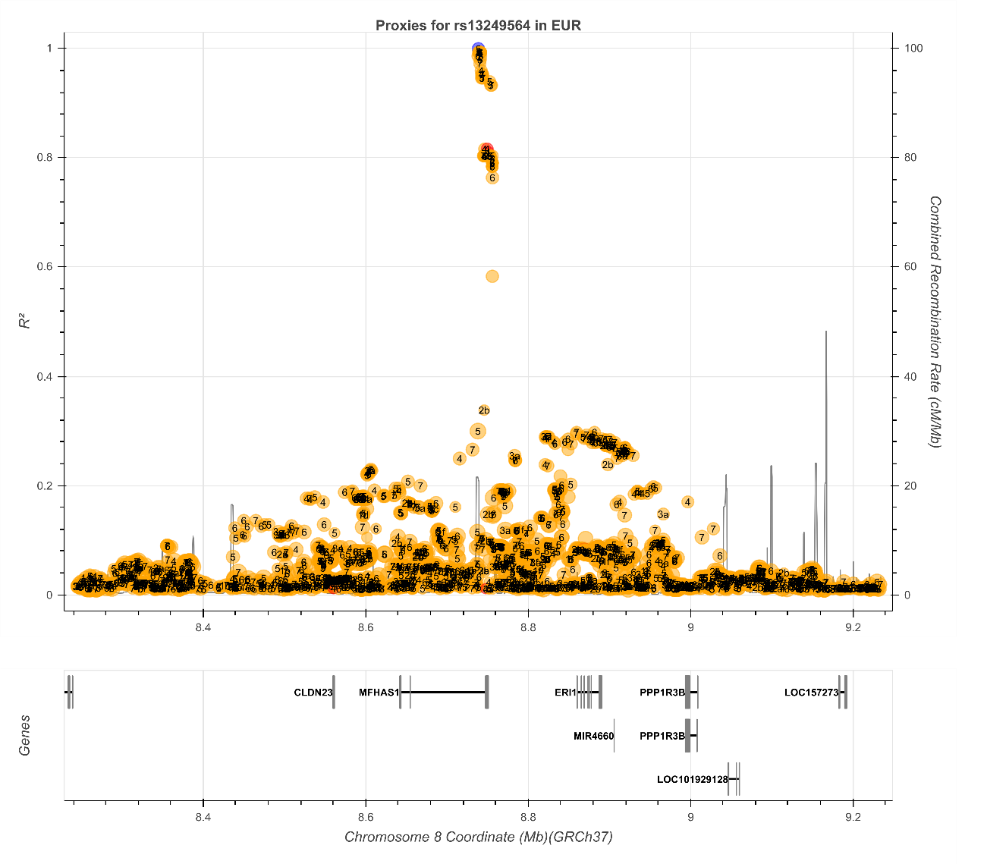


Figure S3. LDLink plot of rs13249564 and its LD proxies at 8p23.1. The horizontal line indicates the r2 threshold (LD=0.8).

**Supplementary** **tables**

| **Table S1**. Relevant demographic and clinical features of study samples analyzed after quality control procedures. | | | | |
| --- | --- | --- | --- | --- |
|  | Cases (n = 343) | Controls (n = 416) | *p*-value* |  |
| Gender (% male)* | 67 | 49 | < 0.0001 |  |
| Age (years, mean ± SD)^#^ | 61 ± 15 | 42 ± 13 | < 0.0001 |  |
| BMI (kg/m^2^, mean ± SD)^#^ | 28 ± 8 | 27 ± 5 | 0.83 |  |
| SAPS (mean ± SD) | 44 ± 13 | NA | NA |  |
| APACHE II (8h) (Median (P_25_–P_75_)) | 20 (16 - 25) | NA | NA |  |
| Comorbidities^$^ (%) | 46 | NA | NA |  |
| ARDS (%) | 36 | NA | NA |  |
| ICU mortality (%) | 36 | NA | NA |  |
| Hospital mortality (%) | 45 | NA | NA |  |
| Sepsis origin (%) |  |  |  |  |
| Pulmonary | 34 | NA | NA |  |
| Non-pulmonary | 66 | NA | NA |  |
| Pathogen (%) |  |  |  |  |
| Gram-positive | 30 | NA | NA |  |
| Gram-negative | 41 | NA | NA |  |
| Others^±^ | 29 | NA | NA |  |
| *Gender comparison was conducted by chi-square test. ^#^Age and body mass index (BMI) were compared using the Mann–Whitney U test.  ^$^Comorbidities include cancer, >80 years, hepatopathy, valvular disease, immunodeficiency, severe brain damage, morbid obesity, chronic disease, autoimmune disease, pregnancy, myopathy, pneumonia, and serious recurrent infections.  ^±^Others include both (Gram-positive and Gram-negative), fungi, virus, and polymicrobial.  APACHE II, Acute Physiology and Chronic Health Evaluation II; ARDS, acute respiratory distress syndrome; BMI, Body Mass Index; ICU, Intensive Care Unit; P25, Percentile 25; P75, Percentile 75; SAPS, Simplified Acute Physiology Score II. | | | | |

| **Table S2**. Association results of the 114 significant positions at 8p23.1 resulting from the admixture mapping study of sepsis. | | | | | |
| --- | --- | --- | --- | --- | --- |
| **Position (hg19)** | **rsID** | **Function** | **Nearest genes** | **OR (95% CI)** | ***p*-value** |
| 8155475 | rs2945891 | intergenic | *FAM86B3P\PRAG1* | 0.50 (0.39 – 0.64) | 1.54E-04 |
| 8171978 | rs13281575 | intergenic | *FAM86B3P\PRAG1* | 0.50 (0.39 – 0.64) | 1.53E-04 |
| 8178223 | rs1548198 | intronic | *PRAG1* | 0.50 (0.39 – 0.64) | 1.51E-04 |
| 8198225 | rs2945913 | intronic | *PRAG1* | 0.50 (0.39 – 0.64) | 1.48E-04 |
| 8207339 | rs17150353 | intronic | *PRAG1* | 0.50 (0.39 – 0.64) | 1.45E-04 |
| 8263945 | rs2979146 | intergenic | *PRAG1\CLDN23* | 0.50 (0.39 – 0.64) | 1.48E-04 |
| 8269691 | rs2945865 | intergenic | *PRAG1\CLDN23* | 0.50 (0.39 – 0.65) | 1.51E-04 |
| 8271252 | rs12680219 | intergenic | *PRAG1\CLDN23* | 0.50 (0.39 – 0.65) | 1.50E-04 |
| 8283667 | rs2945861 | intergenic | *PRAG1\CLDN23* | 0.50 (0.39 – 0.65) | 1.47E-04 |
| 8298310 | rs35930202 | intergenic | *PRAG1\CLDN23* | 0.50 (0.39 – 0.65) | 1.46E-04 |
| 8304502 | rs2921077 | intergenic | *PRAG1\CLDN23* | 0.50 (0.39 – 0.64) | 1.45E-04 |
| 8320029 | rs2979179 | intergenic | *PRAG1\CLDN23* | 0.50 (0.39 – 0.65) | 1.46E-04 |
| 8337819 | rs2979191 | intergenic | *PRAG1\CLDN23* | 0.50 (0.39 – 0.65) | 1.44E-04 |
| 8346690 | rs2976909 | intergenic | *PRAG1\CLDN23* | 0.50 (0.39 – 0.65) | 1.44E-04 |
| 8347248 | rs2921026 | intergenic | *PRAG1\CLDN23* | 0.50 (0.39 – 0.65) | 1.44E-04 |
| 8347822 | rs2980747 | intergenic | *PRAG1\CLDN23* | 0.50 (0.39 – 0.65) | 1.44E-04 |
| 8354436 | rs4840344 | intergenic | *PRAG1\CLDN23* | 0.50 (0.39 – 0.65) | 1.44E-04 |
| 8354546 | rs2921001 | intergenic | *PRAG1\CLDN23* | 0.50 (0.39 – 0.65) | 1.44E-04 |
| 8359387 | rs2921092 | intergenic | *PRAG1\CLDN23* | 0.50 (0.39 – 0.65) | 1.44E-04 |
| 8362710 | rs11783173 | intergenic | *PRAG1\CLDN23* | 0.50 (0.39 – 0.65) | 1.43E-04 |
| 8374374 | rs4639513 | intergenic | *PRAG1\CLDN23* | 0.50 (0.39 – 0.65) | 1.45E-04 |
| 8378925 | rs7822872 | intergenic | *PRAG1\CLDN23* | 0.50 (0.39 – 0.65) | 1.46E-04 |
| 8382374 | rs17616779 | intergenic | *PRAG1\CLDN23* | 0.50 (0.39 – 0.65) | 1.46E-04 |
| 8388447 | rs7001723 | intergenic | *PRAG1\CLDN23* | 0.50 (0.39 – 0.65) | 1.47E-04 |
| 8388493 | rs11991607 | intergenic | *PRAG1\CLDN23* | 0.50 (0.39 – 0.65) | 1.48E-04 |
| 8460285 | rs17154314 | intergenic | *PRAG1\CLDN23* | 0.50 (0.39 – 0.65) | 1.48E-04 |
| 8485705 | rs2140243 | intergenic | *PRAG1\CLDN23* | 0.51 (0.39 – 0.65) | 1.49E-04 |
| 8500562 | rs17627019 | intergenic | *PRAG1\CLDN23* | 0.51 (0.39 – 0.65) | 1.51E-04 |
| 8506501 | rs10085952 | intergenic | *PRAG1\CLDN23* | 0.51 (0.40 – 0.65) | 1.53E-04 |
| 8514059 | rs17627505 | intergenic | *PRAG1\CLDN23* | 0.51 (0.40 – 0.65) | 1.55E-04 |
| 8540070 | rs6994742 | intergenic | *PRAG1\CLDN23* | 0.51 (0.40 – 0.65) | 1.60E-04 |
| 8577361 | rs1703974 | intergenic | *CLDN23\MFHAS1* | 0.51 (0.40 – 0.65) | 1.61E-04 |
| 8584344 | rs777707 | intergenic | *CLDN23\MFHAS1* | 0.51 (0.40 – 0.65) | 1.62E-04 |
| 8589687 | rs10903310 | intergenic | *CLDN23\MFHAS1* | 0.51 (0.40 – 0.65) | 1.63E-04 |
| 8589783 | rs11781985 | intergenic | *CLDN23\MFHAS1* | 0.51 (0.40 – 0.65) | 1.64E-04 |
| 8597071 | rs6601729 | intergenic | *CLDN23\MFHAS1* | 0.51 (0.40 – 0.65) | 1.64E-04 |
| 8615874 | rs17697237 | intergenic | *CLDN23\MFHAS1* | 0.51 (0.40 – 0.65) | 1.64E-04 |
| 8619322 | rs638703 | intergenic | *CLDN23\MFHAS1* | 0.51 (0.40 – 0.65) | 1.64E-04 |
| 8623637 | rs686189 | intergenic | *CLDN23\MFHAS1* | 0.51 (0.40 – 0.65) | 1.64E-04 |
| 8641259 | rs11906 | 3’UTR | *MFHAS1* | 0.51 (0.40 – 0.65) | 1.64E-04 |
| 8658991 | rs10096881 | intronic | *MFHAS1* | 0.51 (0.40 – 0.65) | 1.65E-04 |
| 8659873 | rs10099808 | intronic | *MFHAS1* | 0.51 (0.40 – 0.65) | 1.66E-04 |
| 8671962 | rs11784052 | intronic | *MFHAS1* | 0.51 (0.40 – 0.65) | 1.67E-04 |
| 8676490 | rs950721 | intronic | *MFHAS1* | 0.51 (0.40 – 0.66) | 1.67E-04 |
| 8690393 | rs11995244 | intronic | *MFHAS1* | 0.51 (0.40 – 0.66) | 1.67E-04 |
| 8722378 | rs10108954 | intronic | *MFHAS1* | 0.51 (0.40 – 0.65) | 1.63E-04 |
| 8726546 | rs11998339 | intronic | *MFHAS1* | 0.51 (0.40 – 0.65) | 1.61E-04 |
| 8729900 | rs10098667 | intronic | *MFHAS1* | 0.51 (0.40 – 0.65) | 1.59E-04 |
| 8735213 | rs10046784 | intronic | *MFHAS1* | 0.51 (0.40 – 0.65) | 1.57E-04 |
| 8739456 | rs396121 | intronic | *MFHAS1* | 0.51 (0.40 – 0.65) | 1.54E-04 |
| 8760138 | rs13282211 | intergenic | *MFHAS1\ERI1* | 0.51 (0.40 – 0.65) | 1.50E-04 |
| 8768104 | rs10109507 | intergenic | *MFHAS1\ERI1* | 0.51 (0.40 – 0.65) | 1.50E-04 |
| 8771402 | rs373204 | intergenic | *MFHAS1\ERI1* | 0.51 (0.40 – 0.65) | 1.49E-04 |
| 8771602 | rs17643342 | intergenic | *MFHAS1\ERI1* | 0.51 (0.40 – 0.65) | 1.49E-04 |
| 8785750 | rs4840374 | intergenic | *MFHAS1\ERI1* | 0.51 (0.40 – 0.65) | 1.49E-04 |
| 8793654 | rs17700611 | intergenic | *MFHAS1\ERI1* | 0.51 (0.40 – 0.65) | 1.47E-04 |
| 8799128 | rs6999615 | intergenic | *MFHAS1\ERI1* | 0.51 (0.40 – 0.65) | 1.48E-04 |
| 8810602 | rs7832398 | intergenic | *MFHAS1\ERI1* | 0.51 (0.40 – 0.65) | 1.48E-04 |
| 8821637 | rs13259143 | intergenic | *MFHAS1\ERI1* | 0.51 (0.40 – 0.65) | 1.48E-04 |
| 8875128 | rs11781203 | intronic | *ERI1* | 0.51 (0.40 – 0.65) | 1.48E-04 |
| 8882491 | rs2979257 | intronic | *ERI1* | 0.51 (0.40 – 0.65) | 1.47E-04 |
| 8890098 | rs1045529 | 3’UTR | *ERI1* | 0.51 (0.40 – 0.65) | 1.47E-04 |
| 8901140 | rs2979269 | intronic | *ERI1* | 0.51 (0.40 – 0.65) | 1.47E-04 |
| 8912705 | rs2100021 | intronic | *ERI1* | 0.51 (0.40 – 0.66) | 1.48E-04 |
| 8928729 | rs10091937 | intronic | *ERI1* | 0.51 (0.40 – 0.66) | 1.50E-04 |
| 8930740 | rs10087078 | intronic | *ERI1* | 0.51 (0.40 – 0.66) | 1.50E-04 |
| 8935247 | rs2979243 | intronic | *ERI1* | 0.51 (0.40 – 0.66) | 1.51E-04 |
| 8937788 | rs6601284 | intronic | *ERI1* | 0.51 (0.40 – 0.66) | 1.51E-04 |
| 8937942 | rs6601285 | intronic | *ERI1* | 0.51 (0.40 – 0.66) | 1.51E-04 |
| 8964985 | rs2898208 | intergenic | *ERI1\PPP1R3B* | 0.51 (0.40 – 0.66) | 1.49E-04 |
| 8966972 | rs2979248 | intergenic | *ERI1\PPP1R3B* | 0.51 (0.40 – 0.66) | 1.50E-04 |
| 8967105 | rs17155244 | intergenic | *ERI1\PPP1R3B* | 0.51 (0.40 – 0.66) | 1.49E-04 |
| 8990577 | rs189798 | intergenic | *ERI1\PPP1R3B* | 0.51 (0.40 – 0.66) | 1.46E-04 |
| 9028261 | rs1530483 | intergenic | *PPP1R3B\LOC101929128* | 0.51 (0.40 – 0.66) | 1.45E-04 |
| 9040306 | rs6999694 | intergenic | *PPP1R3B\LOC101929128* | 0.51 (0.40 – 0.66) | 1.45E-04 |
| 9046312 | rs1968853 | upstream | *LOC101929128* | 0.51 (0.40 – 0.66) | 1.40E-04 |
| 9053914 | rs17155367 | ncRNA_intronic | *LOC101929128* | 0.51 (0.40 – 0.66) | 1.40E-04 |
| 9063027 | rs11779181 | intergenic | *LOC101929128\LOC157273* | 0.51 (0.40 – 0.66) | 1.40E-04 |
| 9070973 | rs2929290 | intergenic | *LOC101929128\LOC157273* | 0.52 (0.40 – 0.66) | 1.41E-04 |
| 9082249 | rs2929457 | intergenic | *LOC101929128\LOC157273* | 0.52 (0.40 – 0.66) | 1.41E-04 |
| 9085355 | rs7841403 | intergenic | *LOC101929128\LOC157273* | 0.52 (0.40 – 0.66) | 1.40E-04 |
| 9103134 | rs17149618 | intergenic | *LOC101929128\LOC157273* | 0.51 (0.40 – 0.66) | 1.37E-04 |
| 9132309 | rs330023 | intergenic | *LOC101929128\LOC157273* | 0.52 (0.40 – 0.66) | 1.37E-04 |
| 9153619 | rs17658270 | intergenic | *LOC101929128\LOC157273* | 0.52 (0.41 – 0.66) | 1.37E-04 |
| 9153807 | rs17149705 | intergenic | *LOC101929128\LOC157273* | 0.52 (0.41 – 0.66) | 1.38E-04 |
| 9154394 | rs17149709 | intergenic | *LOC101929128\LOC157273* | 0.52 (0.41 – 0.66) | 1.40E-04 |
| 9154537 | rs10109698 | intergenic | *LOC101929128\LOC157273* | 0.52 (0.41 – 0.66) | 1.40E-04 |
| 9154694 | rs7820048 | intergenic | *LOC101929128\LOC157273* | 0.52 (0.41 – 0.66) | 1.40E-04 |
| 9165654 | rs427731 | intergenic | *LOC101929128\LOC157273* | 0.52 (0.41 – 0.66) | 1.40E-04 |
| 9175433 | rs13279173 | intergenic | *LOC101929128\LOC157273* | 0.52 (0.41 – 0.66) | 1.40E-04 |
| 9178921 | rs12543276 | intergenic | *LOC101929128\LOC157273* | 0.52 (0.41 – 0.66) | 1.40E-04 |
| 9193501 | rs6999153 | downstream | *LOC157273* | 0.52 (0.41 – 0.66) | 1.42E-04 |
| 9197661 | rs958374 | intergenic | *LOC157273\TNKS* | 0.52 (0.41 – 0.66) | 1.41E-04 |
| 9200251 | rs4332138 | intergenic | *LOC157273\TNKS* | 0.52 (0.41 – 0.66) | 1.41E-04 |
| 9200472 | rs6990912 | intergenic | *LOC157273\TNKS* | 0.52 (0.41 – 0.66) | 1.41E-04 |
| 9201003 | rs2199402 | intergenic | *LOC157273\TNKS* | 0.52 (0.41 – 0.66) | 1.43E-04 |
| 9204425 | rs9329185 | intergenic | *LOC157273\TNKS* | 0.52 (0.41 – 0.66) | 1.44E-04 |
| 9224139 | rs7834497 | intergenic | *LOC157273\TNKS* | 0.52 (0.41 – 0.66) | 1.46E-04 |
| 9224907 | rs11784552 | intergenic | *LOC157273\TNKS* | 0.52 (0.41 – 0.66) | 1.48E-04 |
| 9239458 | rs6601302 | intergenic | *LOC157273\TNKS* | 0.52 (0.41 – 0.66) | 1.50E-04 |
| 9250346 | rs922053 | intergenic | *LOC157273\TNKS* | 0.52 (0.41 – 0.66) | 1.55E-04 |
| 9256631 | rs17729883 | intergenic | *LOC157273\TNKS* | 0.52 (0.41 – 0.66) | 1.55E-04 |
| 9264711 | rs6601306 | intergenic | *LOC157273\TNKS* | 0.52 (0.41 – 0.66) | 1.56E-04 |
| 9267251 | rs2409615 | intergenic | *LOC157273\TNKS* | 0.52 (0.41 – 0.66) | 1.58E-04 |
| 9276163 | rs17730481 | intergenic | *LOC157273\TNKS* | 0.52 (0.41 – 0.66) | 1.57E-04 |
| 9280516 | rs17663071 | intergenic | *LOC157273\TNKS* | 0.52 (0.41 – 0.66) | 1.58E-04 |
| 9288937 | rs17663167 | intergenic | *LOC157273\TNKS* | 0.52 (0.41 – 0.66) | 1.64E-04 |
| 9290753 | rs7006443 | intergenic | *LOC157273\TNKS* | 0.52 (0.41 – 0.67) | 1.68E-04 |
| 9294503 | rs11997395 | intergenic | *LOC157273\TNKS* | 0.52 (0.41 – 0.67) | 1.71E-04 |
| 9294638 | rs1458939 | intergenic | *LOC157273\TNKS* | 0.52 (0.41 – 0.67) | 1.71E-04 |
| 9305999 | rs1466338 | intergenic | *LOC157273\TNKS* | 0.52 (0.41 – 0.67) | 1.71E-04 |
| 9310458 | rs13253327 | intergenic | *LOC157273\TNKS* | 0.52 (0.41 – 0.67) | 1.72E-04 |
| 9312131 | rs11777274 | intergenic | *LOC157273\TNKS* | 0.52 (0.41 – 0.67) | 1.70E-04 |
| 9318404 | rs17150087 | intergenic | *LOC157273\TNKS* | 0.53 (0.41 – 0.67) | 1.75E-04 |
| In bold, the position with strongest significance. | | | | | |

| Table S3. Functional annotation of the top hit (rs13249564) at *MFHAS1 gene and the rs7820910.* | | | |
| --- | --- | --- | --- |
| Chromosome location | Chr8: 8738868 (rs13249564) | | Chr8: 9826340 (rs7820910) |
| Nearest gene | *MFHAS1* (intronic) | | LINC00599 and *MSRA* (intergenic) |
| RegulomeDB (category, score) | TF binding + Dnase peak (4, 0.60906) | | TF binding or DNase peak (5, 0.183) |
| Enhancer histone marks | **H3K4me1**: LNG.A549.ETOH002.CNCR, FAT.ADIP.DR.MSC, FAT.ADIP.NUC, VAS.AOR, BRN.ANG.GYR, BRN.ANT.CAUD, BRN.CING.GYR, BRN.GRM.MTRX, BRN.HIPP.MID, BRN.INF.TMP, BRN.SUB.NIG, BRN.DL.PRFRNTL.CRTX, BRST.HMEC.35, GI.CLN.SM.MUS, GI.CLN.MUC, BLD.DND41.CNCR, GI.DUO.MUC, GI.DUO.SM.MUS, ESC.I3, GI.ESO, ESC.4STAR, BRN.FET.F, BRN.FET.M, GI.L.INT.FET, GI.S.INT.FET, MUS.TRNK.FET, GI.STMC.FET, THYM.FET, SKIN.PEN.FRSK.KER.02, SKIN.PEN.FRSK.KER.03, GI.STMC.GAST, BLD.GM12878, ESC.H1, ESDR.H1.MSC, ESDR.H1.NEUR.PROG, ESC.H9, ESDR.CD184.ENDO, ESDR.CD56.ECTO, ESDR.CD56.MESO, BRST.HMEC, MUS.HSMMT, ESC.HUES48, ESC.HUES6, ESC.HUES64, LNG.IMR90, IPSC.DF.19.11, IPSC.DF.6.9, IPSC.15b, IPSC.18, IPSC.20B, HRT.VENT.L, LIV.ADLT, LNG, MUS.SAT, BRN.NHA, SKIN.NHDFAD, SKIN.NHEK, LNG.NHLF, BONE.OSTEO, OVRY, PANC, BLD.MOB.CD34.PC.F, BLD.MOB.CD34.PC.M, BLD.CD34.CC, BLD.CD14.PC, BLD.PER.MONUC.PC, BLD.CD56.PC, BLD.CD15.PC, BLD.CD4.CD25M.IL17P.PL.TPC, BLD.CD4.CD25M.TPC, BLD.CD4.CD25M.IL17M.PL.TPC, BLD.CD4.CD25M.CD45RO.MPC, BLD.CD4.MPC, BLD.CD4.NPC, BLD.CD4.CD25M.CD45RA.NPC, BLD.CD4.CD25.CD127M.TREGPC, MUS.PSOAS, GI.RECT.MUC.29, GI.RECT.MUC.31, GI.RECT.SM.MUS, HRT.ATR.R, HRT.VNT.R, GI.CLN.SIG, MUS.SKLT.F, MUS.SKLT.M, GI.S.INT, SPLN, GI.STMC.MUC, GI.STMC.MUS, THYM | | **H3K4me1**: BRST.HMEC.35  **H3K27ac**: IPSC.20B, BRN.INF.TMP, BRN.ANG.GYR, BRN.DL.PRFRNTL.CRTX |
| Promoter histone marks | **H3K4me3**: GI.STMC.MUS, IPSC.20B, BLD.CD4.CD25M.IL17M.PL.TPC, BLD.CD4.CD25M.IL17P.PL.TPC, BLD.DND41.CNCR, BRN.ANT.CAUD, MUS.SKLT.F, MUS.SKLT.M, GI.RECT.MUC.31, KID.FET | **H3K27ac**: VAS.AOR, BRN.ANG.GYR, BRN.ANT.CAUD, BRN.HIPP.MID, BRN.INF.TMP, BRN.DL.PRFRNTL.CRTX, GI.CLN.SM.MUS, GI.CLN.MUC, BLD.DND41.CNCR, GI.DUO.SM.MUS, GI.ESO, GI.S.INT.FET, GI.STMC.FET, THYM.FET, SKIN.PEN.FRSK.KER.03, ESDR.H1.BMP4.MESO, ESC.H1, ESC.H9, ESDR.CD56.ECTO, ESC.HUES48, ESC.HUES6, ESC.HUES64, IPSC.DF.19.11, IPSC.20B, LNG, SKIN.NHDFAD, SKIN.NHEK, BONE.OSTEO, BLD.CD4.CD25M.IL17P.PL.TPC, BLD.CD4.CD25M.IL17M.PL.TPC, BLD.CD4.CD25M.CD45RO.MPC, LD.CD4.MPC, BLD.CD4.CD25.CD127M.TREGPC, GI.RECT.MUC.29, GI.RECT.MUC.31, GI.RECT.SM.MUS, HRT.VNT.R, GI.CLN.SIG, MUS.SKLT.F, GI.S.INT, GI.STMC.MUS | **H3K4me3**: BRST.HMEC.35 |
| DNAse | Fetal Brain Female, Fetal Brain Male, Fetal Muscle Trunk, Fetal Lung | H3K9ac: IPSC.20B, BRN.ANG.GYR, ESC.HUES48, IPSC.15b, BLD.CD8.NPC, GI.DUO.MUC, LIV.ADLT | None |
| Altered regulatory motif | None | | HNF4,NF-I |
| Proteins bound | None | | None |
| CHiCP (score) | Mifsud et al dataset (CD34 and lymphoblastoid cell lines): Interaction with several genes (*ERI1*, score = 10.10; *CLDN23*, score = 9.51; *TNKS*, score = 7.94), pseudogene (RP11-62H7.2, score = 10.96) and lincRNA (RP11-10A14.6, score = 9.38). | | None |
| eQTLs | 14 significant eQTLs were found for this variant in different tissues: Skin - Sun Exposed (Lower leg), Heart - Atrial Appendage, Brain – Cortex, Cells - Cultured fibroblasts, Adipose – Subcutaneous, Brain - Caudate (basal ganglia), Brain - Nucleus accumbens (basal ganglia), Skin - Not Sun Exposed (Suprapubic), Brain – Cerebellum, Thyroid, Artery – Aorta, Cells - Cultured fibroblasts, Muscle - Skeletal | | 2 significant eQTLs were found for this variant in cells - cultured fibroblasts and in Brain - Cerebellar Hemisphere |
| Score CAPE dsQTL >0.5 | IMR90 fetal lung fibroblasts Cell Line, HSMM Skeletal Muscle Myoblasts Cells, Foreskin Fibroblast Primary Cells skin02, NHDF-Ad Adult Dermal Fibroblast Primary Cells, Gastric, HSMM cell derived Skeletal Muscle Myotubes Cells, A549 EtOH 0.02pct Lung Carcinoma Cell Line, Psoas Muscle, NHLF Lung Fibroblast Primary Cells. | | None |
| Score CAPE eQTL >0.5 | H9 Cells, iPS DF 6.9 Cells, Foreskin Fibroblast Primary Cells skin01, Foreskin Melanocyte Primary Cells skin01, NH-A Astrocytes Primary Cells, Fetal Muscle Trunk, Fetal Adrenal Gland, iPS DF 19.11 Cells, Placenta, Primary T cells from peripheral blood, Primary B cells from peripheral blood, Monocytes-CD14+ RO01746 Primary Cells. | | None |
| BLD.CD4.CD25M.IL17M.PL.TPC, Primary T helper cells PMA-I stimulated; BLD.CD4.CD25M.IL17P.PL.TPC, Primary T helper 17 cells PMA-I stimulated; BLD.CD8.NPC, Primary T CD8+ naive cells from peripheral blood; BLD.DND41.CNCR, Dnd41 TCell Leukemia Cell Line; BRN.ANG.GYR, Brain Angular Gyrus; BRN.ANT.CAUD, Brain Anterior Caudate; CAPE, cellular dependent deactivating mutations; CD34, human hematopoietic progenitor cell line; CHiCP, capture HiC plotter; dsQTL, DNase I sensitivity quantitative trait loci; eQTL, expression quantitative trait loci; ESC.HUES48, HUES48 Cells; GI.DUO.MUC, Duodenum Mucosa; GI.RECT.MUC.31, Rectal Mucosa Donor 31; IPSC.15b, iPS-15b Cells; IPSC.20B, iPS-20b Cells; KID.FET, Fetal Kidney; LIV.ADLT, Liver; MUS.SKLT.F, Skeletal Muscle Female; MUS.SKLT.M, Skeletal Muscle Male; TF, transcription factor; BLD.CD14.PC, Primary monocytes from peripheral blood; BLD.CD15.PC, Primary neutrophils from peripheral blood; BLD.CD34.CC, Primary hematopoietic stem cells short term culture; BLD.CD4.CD25.CD127M.TREGPC, Primary T regulatory cells from peripheral blood; BLD.CD4.CD25M.CD45RA.NPC, Primary T helper naive cells from peripheral blood; BLD.CD4.CD25M.CD45RO.MPC, Primary T helper memory cells from peripheral blood 1; BLD.CD4.CD25M.IL17M.PL.TPC, Primary T helper cells PMA-I stimulated; BLD.CD4.CD25M.IL17P.PL.TPC, Primary T helper 17 cells PMA-I stimulated; BLD.CD4.CD25M.TPC, Primary T helper cells from peripheral blood; BLD.CD4.MPC, Primary T helper memory cells from peripheral blood 2; BLD.CD4.NPC, Primary T helper naive cells from peripheral blood; BLD.CD56.PC, Primary Natural Killer cells from peripheral blood; BLD.DND41.CNCR, Dnd41 TCell Leukemia Cell Line; BLD.GM12878, GM12878 Lymphoblastoid Cells; BLD.MOB.CD34.PC.F, Primary hematopoietic stem cells G-CSF-mobilized Female; BLD.MOB.CD34.PC.M, Primary hematopoietic stem cells G-CSF-mobilized Male; BLD.PER.MONUC.PC, Primary mononuclear cells from peripheral blood; BONE.OSTEO, Osteoblast Primary Cells; BRN.ANG.GYR, Brain Angular Gyrus; BRN.ANT.CAUD, Brain Anterior Caudate; BRN.CING.GYR, Brain Cingulate Gyrus; BRN.DL.PRFRNTL.CRTX, Brain_Dorsolateral_Prefrontal_Cortex; BRN.FET.F, Fetal Brain Female; BRN.FET.M, Fetal Brain Male; BRN.GRM.MTRX, Brain Germinal Matrix; BRN.HIPP.MID, Brain Hippocampus Middle; BRN.INF.TMP, Brain Inferior Temporal Lobe; BRN.NHA, NH-A Astrocytes Primary Cells; BRN.SUB.NIG, Brain Substantia Nigra; BRST.HMEC, HMEC Mammary Epithelial Primary Cells; BRST.HMEC.35, Breast variant Human Mammary Epithelial Cells (vHMEC); ESC.4STAR, ES-UCSF4 Cells; ESC.H1, H1 Cells; ESC.H9, H9 Cells; ESC.HUES48, HUES48 Cells; ESC.HUES6, HUES6 Cells; ESC.HUES64, HUES64 Cells; ESC.I3, ES-I3 Cells; ESDR.CD184.ENDO, hESC Derived CD184+ Endoderm Cultured Cells; ESDR.CD56.ECTO, hESC Derived CD56+ Ectoderm Cultured Cells; ESDR.CD56.MESO, hESC Derived CD56+ Mesoderm Cultured Cells; ESDR.H1.BMP4.MESO, H1 BMP4 Derived Mesendoderm Cultured Cells; ESDR.H1.MSC, H1 Derived Mesenchymal Stem Cells; ESDR.H1.NEUR.PROG, H1 Derived Neuronal Progenitor Cultured Cells; FAT.ADIP.DR.MSC, Adipose Derived Mesenchymal Stem Cell Cultured Cells; FAT.ADIP.NUC, Adipose Nuclei; GI.CLN.MUC, Colonic Mucosa; GI.CLN.SIG, Sigmoid Colon; GI.CLN.SM.MUS, Colon Smooth Muscle; GI.DUO.MUC, Duodenum Mucosa; GI.DUO.SM.MUS, Duodenum Smooth Muscle; GI.ESO, Esophagus; GI.L.INT.FET, Fetal Intestine Large; GI.RECT.MUC.29, Rectal Mucosa Donor 29; GI.RECT.MUC.31, Rectal Mucosa Donor 31; GI.RECT.SM.MUS, Rectal Smooth Muscle; GI.S.INT, Small Intestine; GI.S.INT.FET, Fetal Intestine Small; GI.STMC.FET, Fetal Stomach; GI.STMC.GAST, Gastric; GI.STMC.MUC, Stomach Mucosa; GI.STMC.MUS, Stomach Smooth Muscle; HRT.ATR.R, Right Atrium; HRT.VENT.L, Left Ventricle; HRT.VNT.R, Right Ventricle; IPSC.15b, iPS-15b Cells; IPSC.18, iPS-18 Cells; IPSC.20B, iPS-20b Cells; IPSC.DF.19.11, iPS DF 19.11 Cells; IPSC.DF.6.9, iPS DF 6.9 Cells; LIV.ADLT, Liver; LNG, Lung; LNG.A549.ETOH002.CNCR, A549 EtOH 0.02pct Lung Carcinoma Cell Line; LNG.IMR90, IMR90 fetal lung fibroblasts Cell Line; LNG.NHLF, NHLF Lung Fibroblast Primary Cells; MUS.HSMMT, HSMM cell derived Skeletal Muscle Myotubes Cells; MUS.PSOAS, Psoas Muscle; MUS.SAT, Muscle Satellite Cultured Cells; MUS.SKLT.F, Skeletal Muscle Female; MUS.SKLT.M, Skeletal Muscle Male; MUS.TRNK.FET, Fetal Muscle Trunk; OVRY, Ovary; PANC, Pancreas; SKIN.NHDFAD, NHDF-Ad Adult Dermal Fibroblast Primary Cells; SKIN.NHEK, NHEK-Epidermal Keratinocyte Primary Cells; SKIN.PEN.FRSK.KER.02, Foreskin Keratinocyte Primary Cells skin02; SKIN.PEN.FRSK.KER.03, Foreskin Keratinocyte Primary Cells skin03; SPLN, Spleen; THYM, Thymus; THYM.FET, Fetal Thymus; VAS.AOR, Aorta | | | |

| **Table S4.** Functional annotation of the top hit (rs13249564) and its 44 proxies (r^2^ >0.8). | | | | | | | | | | | |
| --- | --- | --- | --- | --- | --- | --- | --- | --- | --- | --- | --- |
| **SNP** | **Location** | **LD (r²)** | **Promoter histone marks** | **Enhancer histone marks** | **DNAse** | **Motifs changed** | **CAPE eQTL** | **CAPE dsQTL** | **RegulomeDB rank** | **Intervar** | **LoFTool score** |
| rs13249564 | intronic | 1.00 | IPSC, BLD, GI | 14 tissues | 4 tissues | N/A marks | 0.386* | 0.242 | 4 | Benign | 0.509 |
| rs10092694 | intronic | 0.81 | N/A | N/A | 14 tissues | RP58 | 0.283 | 0.198 | 4 | Benign | 0.509 |
| rs10107645 | intronic | 0.80 | N/A | N/A | SKIN,BRN | 7 altered motifs | 0.102 | 0.295 | 3a | Benign | 0.509 |
| rs1062988 | coding | 0.81 | N/A | N/A | 34 tissues | ELF1,Ets,GCNF | 0.017 | 0.838 | 4 | Benign | 0.509 |
| rs10661736 | None | 0.93 | N/A | N/A | N/A | Irf,TATA | N/A | N/A | 7 | VUS | 0.509 |
| rs111699601 | intronic | 0.96 | GI | 15 tissues | N/A | Nr2f2,TR4,Zbtb12 | 0.259 | 0.105 | 3a | Benign | 0.509 |
| rs13254041 | intronic | 0.92 | ESDR, BLD | 16 tissues | IPSC | STAT | 0.135 | 0.355 | 5 | Benign | 0.509 |
| rs13260641 | intronic | 0.99 | BLD | 5 tissues | N/A | NF-I,Roaz | 0.168 | 0.535 | 5 | Benign | 0.509 |
| rs13263327 | intronic | 0.93 | ESDR, BLD, BRN | 14 tissues | BLD | BDP1 | 0.254 | 0.060 | 4 | Benign | 0.509 |
| rs13265597 | intronic | 0.99 | BLD | 5 tissues | N/A | 14 altered motifs | 0.236 | 0.323 | 6 | Benign | 0.509 |
| rs13268804 | intronic | 0.99 | N/A | 5 tissues | N/A | N/A | 0.165 | 0.107 | 5 | Benign | 0.509 |
| rs13276873 | intergenic | 0.80 | N/A | LNG | 6 tissues | 4 altered motifs | 0.609 | 0.225 | 2b | Benign | 0.509 |
| rs28626220 | intronic | 0.99 | N/A | 6 tissues | N/A | 4 altered motifs | 0.091 | 0.142 | 7 | Benign | 0.509 |
| rs28711612 | intergenic | 0.80 | N/A | N/A | N/A | N/A | 0.027 | 0.134 | 5 | Benign | 0.509 |
| rs34315433 | intronic | 0.97 | N/A | ESC, IPSC, BLD | ESC | GCNF,YY1 | 0.103 | 0.210 | 7 | Benign | 0.509 |
| rs34470797 | intronic | 0.97 | N/A | ESC, IPSC, BLD | N/A | HNF4,Nkx2,RXRA | 0.103 | 0.574 | 6 | Benign | 0.509 |
| rs34695890 | intronic | 0.97 | BLD | ESC, IPSC, BLD | ESC,ESC | 5 altered motifs | 0.031 | 0.474 | 4 | Benign | 0.509 |
| rs35001899 | None | 0.80 | N/A | 7 tissues | SKIN,THYM,BLD | 10 altered motifs | N/A | N/A | 4 | VUS | 0.509 |
| rs35005822 | intronic | 0.99 | IPSC | 12 tissues | N/A | E2A,TBX5,ZEB1 | 0.494* | 0.222 | 4 | Benign | 0.509 |
| rs35060319 | intronic | 0.97 | N/A | ESC, IPSC, BLD | N/A | Pax-5 | 0.272 | 0.756 | 5 | Benign | 0.509 |
| rs35400846 | intergenic | 0.80 | N/A | 6 tissues | N/A | 11 altered motifs | 0.151 | 0.246 | 2b | Benign | 0.509 |
| rs35493619 | intronic | 0.93 | 5 tissues | 19 tissues | ESC,BLD | 7 altered motifs | N/A | N/A | 4 | VUS | 0.509 |
| rs35736441 | intronic | 0.80 | 19 tissues | 8 tissues | 5 tissues | ATF3 | 0.128 | 0.177 | 4 | Benign | 0.509 |
| rs35820021 | intergenic | 0.80 | N/A | 7 tissues | 6 tissues | 4 altered motifs | 0.680 | 0.146 | 2b | Benign | 0.509 |
| rs36025219 | intergenic | 0.80 | N/A | 7 tissues | 7 tissues | BDP1 | 0.422* | 0.175 | 3a | Benign | 0.509 |
| rs36045355 | intronic | 0.88 | ESDR, BLD, BRN | 9 tissues | N/A | 6 altered motifs | 0.061 | 0.412 | 4 | Benign | 0.509 |
| rs36061325 | intergenic | 0.80 | N/A | BLD | KID | NRSF | 0.421* | 0.038 | 4 | Benign | 0.509 |
| rs3748141 | coding | 0.81 | 21 tissues | 6 tissues | 36 tissues | GR,T3R | 0.113 | 0.622 | 4 | Benign | 0.509 |
| rs3762032 | intergenic | 0.91 | N/A | 9 tissues | BLD,BLD | 4 altered motifs | 0.552* | 0.083 | 3a | Benign | 0.509 |
| rs4840373 | intergenic | 0.9 | N/A | N/A | N/A | 12 altered motifs | 0.482* | 0.074 | 5 | Benign | 0.509 |
| rs4841061 | intronic | 0.99 | IPSC | 10 tissues | N/A | N/A | 0.267 | 0.152 | 5 | Benign | 0.509 |
| rs4841062 | intronic | 0.99 | IPSC | 10 tissues | N/A | Esr2,Sox,p300 | 0.243 | 0.246 | 3a | Benign | 0.509 |
| rs4841063 | intronic | 0.99 | N/A | ESC, IPSC, BLD | N/A | 6 altered motifs | 0.355 | 0.644 | 3a | Benign | 0.509 |
| rs4841064 | intronic | 0.97 | N/A | 5 tissues | SKIN | N/A | 0.137 | 0.273 | 4 | Benign | 0.509 |
| rs4841065 | intronic | 0.97 | N/A | 5 tissues | SKIN | N/A | 0.135 | 0.292 | 4 | Benign | 0.509 |
| rs4841066 | intronic | 0.94 | N/A | 5 tissues | N/A | 11 altered motifs | 0.087 | 0.423 | 5 | Benign | 0.509 |
| rs4841067 | intronic | 0.80 | 19 tissues | 7 tissues | 5 tissues | GR | 0.635* | 0.030 | 4 | Benign | 0.509 |
| rs6601267 | intronic | 0.80 | 5 tissues | 9 tissues | N/A | 6 altered motifs | 0.088 | 0.035 | 7 | Benign | 0.509 |
| rs7008835 | intronic | 0.80 | 6 tissues | 7 tissues | N/A | DMRT4,Pax-3 | 0.080 | 0.153 | 7 | Benign | 0.509 |
| rs7013902 | intronic | 0.80 | 19 tissues | 8 tissues | 5 tissues | 4 altered motifs | 0.201 | 0.267 | 4 | Benign | 0.509 |
| rs71219098 | None | 0.80 | 5 tissues | 13 tissues | PANC | Foxa,Pax-5,p300 | N/A | N/A | 4 | VUS | 0.509 |
| rs73192215 | intronic | 0.93 | 6 tissues | 18 tissues | 12 tissues | 4 altered motifs | 0.192 | 0.231 | 4 | Benign | 0.509 |
| rs73192215 | intronic | 0.95 | 6 tissues | 18 tissues | 12 tissues | 4 altered motifs | 0.192 | 0.231 | 4 | Benign | 0.509 |
| rs7844068 | intronic | 0.93 | 10 tissues | 19 tissues | ESC,ESC,IPSC | Irf | 0.446* | 0.117 | 4 | Benign | 0.509 |
| rs9942753 | intronic | 0.93 | ESDR, BLD | 13 tissues | GI | AP-1,Foxa,RORalpha1 | 0.040 | 0.217 | 4 | Benign | 0.509 |
| CAPE, CellulAr dePendent dEactivating; eQTL, expression quantitative trait loci; dsQTL, DNase I sensitivity quantitative trait loci; LD, Linkage disequilibrium; VUS, variant of uncertain significance; ESDR, hesc derived; GI, Gastrointestinal; BLD, Blood; BRN, Brain; IPSC, Induced pluripotent stem cells; LNG, Lung; ESC, Embryonic stem cells; THYM, Thyme; KID, Kidney; PANC, Pancreas; FOXA1, Forkhead box protein A1; FOXA2, Forkhead box protein A2; HNF4G, Hepatocyte nuclear factor 4 gamma; CCNT2, Cyclin T2; E2F6, E2F Transcription Factor 6; USF1, Upstream Transcription Factor 1; CEBPB, CCAAT Enhancer Binding Protein Beta; POL2, RNA polymerase II; TAF1, TATA-Box Binding Protein Associated Factor 1; RP58, Zinc Finger Protein 513; ELF1, E74 Like ETS Transcription Factor 1; Ets ETS Proto-Oncogene 1, Transcription Factor; GCNF, Nuclear Receptor Subfamily 6 Group A Member 1; Irf, Interferon Regulatory Factor; TATA, TATA box; Nr2f2, Nuclear Receptor Subfamily 2 Group F Member 2; TR4, Testicular receptor 4; Zbtb12, Zinc Finger And BTB Domain Containing 12; STAT, Signal Transducer And Activator Of Transcription; NF-I, Nuclear factor I; Roaz, zinc finger protein 423; BDP1, B Double Prime 1, Subunit Of RNA Polymerase III Transcription; YY1, YY1 Transcription Factor; HNF4, Hepatocyte Nuclear Factor 4; Nkx2, NK2 Homeobox 1; RXRA, Retinoid X Receptor Alpha; E2A, Transcription Factor 3; TBX5, T-Box Transcription Factor 5; ZEB1, Zinc Finger E-Box Binding Homeobox 1; Pax-5, Paired Box 5; ATF3, Activating Transcription Factor 3; NRSF, Neuron-Restrictive Silencer Factor; T3R, T3 receptors ; Esr2, Estrogen Receptor 2; Sox, SRY-related HMG-box; p300, E1A Binding Protein P300; GR, Glucocorticoid receptor; DMRT4, DM-related transcription factor 4; Pax-3, Paired Box 3; Foxa, Forkhead box protein A; AP-1, Activator protein 1; RORalpha1, Retinoid-related orphan receptor alpha1.  *Relevant CAPE eQTL score. | | | | | | | | | | | |

| **Table S5.** Meta-analysis results of differential gene expression analysis of the genes in the significant admixture mapping region (plus 1 Mb on each side). | | |
| --- | --- | --- |
| **Gene** | **q-value** | **Fold-change mean** |
| *DEFA4* | 9.40E-18 | 0.563 |
| *TNKS* | 1.08E-12 | -0.066 |
| *AGPAT5* | 7.46E-11 | -0.190 |
| LINC00965 | 1.94E-08 | -0.105 |
| *BLK* | 6.03E-08 | -0.170 |
| *XKR6* | 1.12E-07 | -0.135 |
| *MCPH1* | 4.34E-07 | -0.106 |
| *DEFT1P* | 1.55E-06 | 0.245 |
| *DEFT1P2* | 1.55E-06 | 0.245 |
| *NEIL2* | 1.85E-06 | -0.095 |
| *MSRA* | 3.54E-06 | 0.098 |
| *PPP1R3B* | 1.41E-05 | 0.079 |
| *CLDN23* | 1.53E-05 | -0.040 |
| *MFHAS1* | 2.38E-05 | -0.082 |
| *DEFA1B* | 5.95E-05 | 3.810* |
| *DEFA3* | 1.41E-04 | 3.750* |
| *SOX7* | 1.86E-04 | -0.039 |
| *ERI1* | 3.47E-04 | 0.125 |
| *DEFA1* | 3.59E-04 | 3.730* |
| *PINX1* | 3.83E-04 | -0.047 |
| *FDFT1* | 0.0027 | -0.060 |
| *DEFA5* | 0.0029 | 0.056 |
| *CTSB* | 0.0032 | 0.095 |
| *FAM86B3P* | 0.0032 | -0.049 |
| *ZNF705G* | 0.0066 | 0.023 |
| *FAM167A* | 0.0067 | -0.042 |
| *SPAG11A* | 0.0130 | 0.036 |
| *DEFB1* | 0.0153 | -0.023 |
| *MTMR9* | 0.0217 | -0.016 |
| *FAM86B1* | 0.0473 | -0.212* |
| *C8orf49* | 0.0576 | 0.071 |
| *DEFA6* | 0.0637 | 0.028 |
| *TDH* | 0.0894 | 0.035 |
| *MIR124-1* | 0.1177 | 0.014 |
| *DEFB106A* | 0.1383 | 0.052 |
| *DEFB106B* | 0.1383 | 0.052 |
| LOC101929269 | 0.1828 | 0.044 |
| *ZNF705D* | 0.2490 | 0.050* |
| *C8orf74* | 0.2500 | -0.020 |
| LOC100129129 | 0.2562 | 0.042 |
| *RP1L1* | 0.2786 | 0.032 |
| *DEFB105A* | 0.2976 | 0.040 |
| *DEFB105B* | 0.2976 | 0.040 |
| LINC00208 | 0.3269 | -0.032 |
| *SPAG11B* | 0.3874 | 0.012 |
| *FAM167A-AS1* | 0.4790 | -0.039* |
| *DEFB107A* | 0.5265 | -0.020 |
| *DEFB107B* | 0.5265 | -0.020 |
| LOC157273 | 0.5269 | 0.022 |
| *ANGPT2* | 0.5834 | 0.005 |
| *XKR5* | 0.5852 | -0.035* |
| *SLC35G5* | 0.6063 | 0.018 |
| *PRSS55* | 0.6102 | 0.016 |
| LOC100287015 | 0.6258 | 0.027 |
| *DEFB4A* | 0.6485 | 0.051* |
| *FAM66D* | 0.6668 | 0.006 |
| *ZNF705B* | 0.7609 | -0.069* |
| *MIR598* | 0.7891 | 0.021* |
| *DEFB103A* | 0.7893 | 0.025* |
| *GATA4* | 0.8095 | 0.002 |
| *USP17L2* | 0.8135 | 0.019* |
| *FAM66A* | 0.8177 | -0.014* |
| *DEFB135* | 0.8385 | 0.014* |
| *PRR23D1* | 0.8431 | 0.016* |
| GS1-24F4.2 | 0.8539 | -0.014 |
| *DEFB104A* | 0.8945 | 0.010 |
| *DEFB104B* | 0.8945 | 0.010 |
| *LOC392196* | 0.9285 | -0.015* |
| *MIR597* | 0.9531 | 0.014* |
| *MIR1322* | 0.9663 | 0.007* |
| *DEFB103B* | 0.9677 | 0.004* |
| *DEFB136* | 0.9908 | 0.001* |
| *Results available only for one of the datasets (GSE32707) | | |
